# The use of dance to improve the health and wellbeing of older adults: A global scoping review of research trials

**DOI:** 10.1371/journal.pone.0311889

**Published:** 2024-10-22

**Authors:** Martha Waugh, Gregory Youdan, Courtney Casale, Rachel Balaban, Emily S. Cross, Dafna Merom

**Affiliations:** 1 School of Health Sciences, Western Sydney University, Sydney, New South Wales, Australia; 2 Department of Theatre Arts and Performance Studies, Brown University, Providence, Rhode Island, United States of America; 3 Department of Dance, The Juilliard School, New York, New York, United States of America; 4 School of Psychological Sciences, Macquarie University, Sydney, New South Wales, Australia; 5 Social Brain Sciences Group, ETH Zürich, Zürich, Switzerland; 6 Warren Alpert Medical School, Brown University, Providence, Rhode Island, United States of America; 7 Translational Health Research Institute, Western Sydney University, Sydney, New South Wales, Australia; Hamasaki Clinic, JAPAN

## Abstract

**Background:**

Dance is a promising health resource for older adults, but empirical evidence remains inconsistent. The lack of synthesised evidence regarding program design, dose, and delivery limits understanding of factors influencing participation and health outcomes. This scoping review aimed to map the scope, range, and effectiveness of dance programs for older people, and identify gaps and opportunities for future research and practice.

**Methods:**

Searches across five databases (September 2023) identified 148 studies evaluating 116 dance programs (≥4 weeks) for older adults (≥55 years, N = 8060), Dance interventions delivered to clinical groups were excluded. Intervention design and delivery were charted against the TIDieR reporting checklist. Program outcomes including adherence, safety, and positive tests were charted into established taxonomies.

**Results:**

Demographic information, program details, and implementation were often insufficiently reported. Participant groups differed by age range, with underserved communities underrepresented. Programs varied extensively in key factors including dose, prospective ‘active ingredients’, delivery approach, facilitator expertise, and class size. While dance was physically safe, adherence rates in older adults are comparable to other community exercise programs. Less than 40% of health assessments showed positive change, with more consistent benefits to physical endurance, strength, and function, moderate impacts on psychosocial health, and limited benefits to cognitive and brain health, and falls and falls risk.

**Conclusion:**

Dance is a meaningful, safe, adaptable, and low-cost health resource for older adults. Key opportunities for advancing research include improved access for underserved groups, program suitability assessments, strategies to support adherence and engagement including theory-informed approaches, and incorporation of participant and practitioner insights. Identification of key ’active ingredients’ and dance program factors may improve understanding of causal pathways and mechanisms to optimise engagement and health impacts. Stronger reporting practices will facilitate comparisons across studies and more robust evidence synthesis. This review provides a critical knowledge foundation to guide future approaches in dance for health and offers reporting recommendations.

## Introduction

Interest in and research exploring the health and wellbeing benefits of arts and cultural activities have grown exponentially over the past few decades [1]. This progress has been accompanied by the increasing integration of arts activities into health and social care [2, 3]. For older adults, dancing is recommended exercise for optimal aging [4], enriched with music, social opportunities, and shared embodied arts experiences [1, 5–7]. Participation in group dance programs enables older people to be learners and creators to support both active aging [8, 9] and creative aging [1, 10], and offers an effective, accessible, economical, and sustainable approach to promoting health into later life.

However, inconsistent outcomes across primary studies and meta-analyses evaluating the health benefits of dancing [e.g., 11–15], coupled with the absence of well-defined theoretical foundations and consistent methodologies [16] have introduced challenges in organising, interpreting, and applying the findings meaningfully. These issues have led to difficulties in formulating recommendations for treatment and optimisation of heath impacts [16–19].

Current efforts in the arts health field are focused on developing approaches and frameworks to guide future research and practice, including the use of arts and culture in public health and mechanisms of change [2, 20, 21]. Evidence synthesis in dance for health, and arts health more generally, has yet to account for differences in activity types, design, and delivery which may impact participation and subsequent health outcomes. Clift and colleagues’ [17] critique of current approaches in arts health research emphasises the lack of attention to the “artistic quality and integrity” [p3] of activities, a lack of specificity in defining activities involved in interventions, and the over-generalisation of findings beyond a target population.

Although several researchers have highlighted issues with study design and risk of bias in dance health research [e.g., 11, 19, 22], empirical investigation of specific dance programs or trials and their differential impact on health outcomes has been limited. A single meta-analysis examining the benefits of dance on cognitive function in older people found a dose-response relationship between dance and global cognition [12]. Additionally, McCrary and colleagues’ [11] umbrella review of the health impacts of music and dance programs without explicit exercise intensity targets synthesised evidence on the activity dose, dance style, and physical intensity. The study reported the broadest positive effects on health for dance programs over 4 weeks, and for aerobic dance, ballroom dance, social dance (4 health domains improved), and Zumba (3 domains improved), and further highlighted variability in physical intensity within and across different dance styles [11].

Receptive and participatory arts activities are considered complex interventions as they have numerous components that interact to generate psychological, biological, social and behavioural processes that can co-produce various health and behavioural outcomes on an individual and group level and will affect people differently [2, 21, 23, 24]. Dance programs delivered live are particularly dynamic, multi-faceted, interactive, and responsive to individuals and groups [19, 24]. Attempting to categorise the component parts of dance programs is undoubtedly reductive. However, to better understand how dancing affects health and wellbeing–the causal processes or mechanisms of action through which health benefits are achieved–and what factors may act as moderators of these processes, it is necessary first to establish the core components of dance for health programs and how they vary.

Group dance programs likely share common factors alongside properties unique to specific dance styles or approaches. Fortin’s [19] narrative overview of dance for health programs in Canada emphasises the diversity of existing programs across dimensions such as context, target populations, expertise of program facilitators, pedagogy and approaches to delivery, content, degree of standardisation, and program goals. Evidence from the broader literature also suggests numerous components of dance programs that may determine the health impacts. For example, physical intensity [8, 11, 25], balance challenges [26, 27], and the degree of motor-skill learning [28, 29] involved in a program may influence outcomes including cardiovascular endurance, physical and cognitive function, falls, falls risk, and falls self-efficacy.

Our literature review of dance for older people found a range of health and social outcomes investigated or targeted for improvement. Connecting dance program components with these outcomes may identify ‘active ingredients’ that directly and differentially contribute to program success across various domains and populations [21, 23]. Determining these components, including their level or dose, may explain inconsistencies in health benefits realised through group dancing, inform program design, delivery, and evaluation, and enable practitioners to tailor programs more effectively to meet the needs of older adults.

A scoping review is the approach best suited to synthesise evidence of intervention (program) types, design, and delivery, alongside program-related health outcomes. Scoping reviews allow researchers to map key features and concepts associated with a research area through a systematic process of literature searching and screening, data charting, and data analysis [30]. This approach can address broad questions related to a field and produce an overview of a topic, identifying gaps and patterns in developing knowledge areas such as arts health.

Prior to conducting this research, the team searched for similar reviews or protocols and relevant frameworks to support the identification, categorisation, and synthesis dance for health programs. We found a single scoping review published in 2023 that synthesised 14 dance programs for adults with neurodevelopmental disabilities, but primarily focused on outcome measures and mechanisms of change with limited program details [31]. Golden and colleagues’ [32] thorough review of the uses of music to manage serious mental illness provided a promising approach to synthesising evidence of populations, studies, and activity types alongside outcomes, which informed our approach to evidence mapping and overall research goals.

Relevant frameworks included the INNATE framework [23] which maps potential ‘active ingredients’ in arts in health activities, in part to facilitate comparisons between arts interventions. INNATE includes 139 distinct ingredients in receptive and active arts activities. Fortin’s [19] appraisal of dance health initiatives also provided details on target populations, facilitators, content, and pedagogy. Our identification of core properties and characteristics of dance programs was guided by Fortin’s review, the INNATE ‘ingredients’ relevant to group dance sessions, and examination of the dance health and adjacent literature.

Arts health researchers have advocated for research methods and future programs of enquiry that are sensitive to the dynamic, responsive, interpersonal, and human qualities of arts activities [16, 19, 32, 33]. Fortin [19] and Golden [32] also recommend developing labels or taxonomies to differentiate distinct activities within an arts health area, to allow for effective organisation, aggregation, and comparison to guide research, policy and arts prescription. This study offers a novel, systematic, in-depth overview of dance for health research, focused on improving health and wellbeing outcomes for older people.

A scoping review was undertaken to understand the use of dance to improve health and wellbeing in older people. The objectives of the scoping review were:

1. To map the scope, variability, and overall effectiveness of dance for health programs designed to improve the health and wellbeing of older adults, from all published research studies;
2. To identify evidence gaps and opportunities; and
3. To formulate recommendations for future research in dance for health to support evidence synthesis and improve research practices, with the overall goal of optimising impact and accessibility

## Materials and methods

### Design and protocol registration

A scoping review was undertaken to map group dance participant and program characteristics, to understand the breadth and distribution of existing evidence of dance for health program efficacy, and to identify gaps in intervention reporting. This review followed the JBI scoping review methodology [30] and was reported in accordance with the PRISMA-ScR Reporting Guidelines [34]. The review protocol was registered in March 2023 with the Open Science Foundation (https://osf.io/zwcu5/). An iterative approach was taken to searching the research literature, refining the inclusion and exclusion criteria, reviewing the studies, and charting the relevant data.

### Eligibility criteria

Studies were included if they evaluated health and/or wellbeing benefits associated with participation in group dance programs ≥4 weeks duration for a general population of older adults ≥55 years and delivered live, either in-person or remotely. All dates, all context and settings, and all geographic locations were considered. The types of evidence sources considered were peer-reviewed, published research trials of any design. During the protocol design, a participant age cutoff of ≥55 years was chosen to ensure the review represented dance studies that deliberately targeted ‘older adults’ as this is a fairly common minimum age in dance trials and is in line with previous dance for health reviews for the older population [e.g., 12, 14, 35].

Dance research studies for specific clinical populations (e.g., Dance for Parkinson’s) were excluded to ensure the content of the dance programs had not been adapted to meet clinical needs, thus maintaining focus on dance sessions suitable and relevant for the general population of older adults. Further exclusion criteria were: Studies of dance programs designed for and delivered to older people with specific clinical, preclinical, or subclinical disease or health conditions or for rehabilitation; Programs where dance was not the primary activity, other movement, meditation, mind-body activities, and physical therapy to music; Nongroup dance activities and programs not delivered live, including dance exergaming, pre-recorded dance classes completed at home, and 1-to-1 dance sessions; Studies not available in English, conference abstracts, unpublished dissertations, and systematic reviews and meta-analyses.

### Search strategy and study selection

Key search terms were identified using the population, concept, context (PCC) criteria for JBI scoping reviews [30]. We searched five databases; MEDLINE (OVID), Embase, CINAHL Plus, PsycINFO, and Web of Science on 27^th^ September 2023. The search strategy for Medline and the PRISMA-ScR reporting checklist is available in S1 Table. The second (final) search removed the term ‘movement therapy’ to reduce irrelevant results. Searches were tested on five pearl articles relevant to the topic of enquiry with all properties specified in the inclusion criteria, which were retained.

The search results were exported into EndNote or Zotero, de-duplicated, and uploaded to Covidence for screening. Relevant systematic reviews and meta-analyses were retained from the search and/or identified by the research team and were hand-searched for citations. Title and abstract screening were conducted in Covidence by blinded pairs of trained reviewers. Discrepancies in applying the inclusion and exclusion criteria were taken to a third reviewer and resolved through discussion when necessary. Discussion included the scope of preclinical and subclinical conditions (e.g., whether to include obesity, metabolic syndrome, mild cognitive impairment, which were all omitted). Full texts were screened for relevance by blinded pairs of trained reviewers, and reason for exclusion was documented.

### Dance trial and program data integration

In many cases, a single trial of a dance program was reported across more than one publication. Throughout data charting, the first author (MW) checked all articles for references to previous publications of the same dance trial or program and cross-referenced author names, protocol registration, clinical trial or funding codes, and basic study and program characteristics with eligible studies sharing similar features. The information was recorded in a separate data file.

After data charting, publications reporting a single trial of a dance program were grouped together and common data were amalgamated for further analysis. The first published study or the paper with the most comprehensive reporting was used to resolve any discrepancies (e.g. in sample size or demographics). We also extracted and integrated all relevant intervention data from registered protocols and other referenced articles (typically previous iterations of the dance program not eligible for the current review).

The cross-referencing procedure identified several publications that potentially reported the same dance trial, but without citation. After each wave of data extraction was completed, MW contacted the corresponding author/s of the aforementioned studies via email and/or other method (e.g., ResearchGate) for confirmation, and followed up again a month later. Some publications were confirmed to report different trials or were not referenced due to close article submission dates. Where authors or research groups did not respond to our queries, MW used the available information to determine whether the publications were most likely to be reporting the same or a different trial. In all, 13 articles were identified without author input as duplicate/multiple/secondary publications of 5 trials.

### Data charting

Data charting was completed in Covidence. To maintain consistency, the data extraction form was accompanied by an instructional guide and was tested on a subsample of articles by two teams of reviewers and revised several times. Data charting was performed by one reviewer per study and all extracted data were checked through again by MW and standardised where required.

Charted data included: 1) Study characteristics (title, authors, year of publication, study design, methods, study aims, sample size); 2) Trial context (global region, country, setting, inclusion and exclusion criteria, target population description, recruitment criteria); 3) Participant characteristics (age, gender, race and ethnicity); 4) Dance program characteristics and delivery (dance style/genre, rationale and development, co-design processes, program structure, physical intensity, challenges and opportunities, standardisation and tailoring, pedagogical methods and approaches to program delivery); 5) Program outcomes reported by authors (program adherence/attendance, safety, outcome measures and results (statistically significant positive effect vs. no effect or statistically significant negative effect); and 6) Comparator types and activities.

### Program characteristic and outcome stratification

To categorise the characteristics and delivery of the dance programs, we used a standardised, well-accepted intervention reporting tool, the Template for Intervention Description and Replication (TIDieR) developed by Hoffmann and colleagues [36]. The TIDieR checklist specifies the following information: 1) Program name and brief description; 2) WHY (rationale, theory, or goal of the program); 3/4) WHAT (physical or information materials and procedures, activities or processes); 5) WHO provided (expertise, background, and training undertaken); 6) HOW (mode of program delivery); 7) WHERE (locations and necessary infrastructure); 8) WHEN and HOW MUCH (schedule, dose, duration, frequency, intensity); 9) TAILORING (program personalisation or adaptation), 10) MODIFICATIONS (changes made to the program); and 11/12) HOW WELL (program as planned vs. actual program delivered, including assessments of program adherence and fidelity).

Stratification of the health and wellbeing outcome domains evaluated in the dance trials was based on the taxonomy developed by Dodd and colleagues [37] for the Core Outcome Measures in Effectiveness Trials (COMET) database of core outcome sets. Qualitative research approaches generally do not evaluate the impact of dancing on a particular health domain or area of functioning. Instead, these methods explore participant experiences and perspectives, which can include perceived health and social impacts for some proportion of participants, which are then interpreted and categorised by researchers. However, for brevity, simplicity, and consistency, only outcomes assessed quantitatively will be included.

It was necessary to adapt and subdivide some of the outcome categories in the COMET taxonomy [37] to align with the outcomes as characterised and most frequently assessed in the dance health trials included for review, which was achieved through discussion by the research team. We further referenced McCrary and colleagues’ organisation of outcome domains included their dance and music health umbrella review [11]. The health domains evaluated in the research studies and included in this review are listed in Table 7. The outcome measures were allocated under the relevant health outcome domain and the total number of tests conducted in each domain and the number of tests demonstrating positive change were recorded. This final step was an addition to the protocol.

### Data collation and synthesis

Data collation was a substantive process which involved: 1) The classification and standardisation of program properties (e.g., the item ‘WHO provided’: number and expertise of facilitators and assistants; what and how much training was provided and to whom; estimated class sizes and student-teacher ratios); and 2) The identification and consolidation of recurring features and characteristics from each TIDieR category. Both procedures were data-driven and validated against the INNATE framework of ‘active ingredients’ [23] to indicate key program components.

Data synthesis took several approaches: 1) Numerical and narrative summaries of the study design, participant characteristics and dance health program characteristics and implementation (Tables 1–3 and 5); 2) Descriptive statistical analysis of program dose, class size and student-teacher ratios, program adherence/attendance, and percentage of statistically significant positive test outcomes in each outcome domain (Tables 4, 6 and 7, Fig 3); and 3) Evidence mapping of dance health trial reporting, colour-coded to visually illustrate the comprehensiveness of reporting across each TIDieR category (Fig 4).

**Table 1:** Dance trials number of participants for each WHO global region.

Global region	Trials		Countries	Participants
	N	%	N	N
Americas	42	37.4	4	2454
Europe	39	33.9	16	3122
Western Pacific	23	20.0	8	1914
Southeast Asia	9	7.8	2	559
Eastern Mediterranean	1	0.9	1	11
Africa	0	0.0	-	-
**Total**	**115**	**100.0**	**31**	**8060**

**Table 2:** Trial setting.

Setting	Trials		Participants
	N	%	N
Community general	78	67.8	5267
Senior/adult day-care facilities	9	7.8	415
Independent living/self-care retirement	4	3.5	649
Aged care/Nursing home	11	9.6	755
Inpatient	1	0.9	313
Outpatient clinic	2	1.7	57
Not reported	10	8.7	604
**Total**	115	100.0	8060

**Table 3:** Style or genre of dance health programs for older adults.

Dance style categories	Programs		People	Dance styles
	N	%	N	
**Folk/National/Social dance**	28	24.1	2154	Chinese square dance, Latin dance, Line dancing, Salsa dance, Samba carnival parade, traditional American, English, Greek, Indonesian, Polish, Caribbean, and Turkish folk dance
**Mixed styles & specialist programs**	27	23.3	1618	Mixed style and specialised programs developed for seniors, DanceSport, Agliando^™^, GERAS dance, Lebed method/Healthy Steps, DMT-informed, Otago falls prevention exercises, cognitive training via robot
**Global South & North performance dance**	24	20.7	1537	Argentine Tango, traditional Thai dance, traditional Chinese dance, Ballet, Contemporary dance, Irish dance, Spanish dance, Tap dance, traditional Korean dance
**Creative dance & dance movement therapy (DMT)**	13	11.2	990	Programs predominately improvisation, guided discovery, and creative tasks, DMT and DMT-informed elements, may include section/s of choregraphed (set) dance exercises
**Aerobic exercise dance**	12	10.3	635	Including gymnastic dance, low impact, specialised dance aerobics programs for seniors, Thai boxing dance, Zumba
**Ballroom dance**	10	8.6	1132	Rock and Roll, Foxtrot, slow and traditional Waltz, Bachata, Cha Cha, Cancan, Salsa, Rumba, Polka, Country, Swing, Bolero, Samba, Forro
**TOTAL** ^1^	**116**	**98.3**	**8096**	**-**

^1^Two dance programs did not specify a program dance style, but the programs and program participants (n = 30) are included in totals

**Table 4:** Dose of group dancing.

Dose	Mean	(SD)	Range	Mode	Median
Session length (mins)	59.0	(16.3)	15–105	60	60
Session frequency (per week)	2.4	(1.4)	1–14	3	2
Program duration (weeks total)^1^	15.8	(12.5)	4–78	12	12
**Total hours**	**37.9**	**(41.5)**	**1.5–234**	**24**	**25**

^1^Scoping review criteria included programs of ≥4 weeks duration only

Note: When session length or session frequency per week varied across the program (e.g. some programs progressively increased the session length) we took the average length/frequency.

**Table 5:** Expertise of dance program facilitators.

Facilitator/s	Trials	
	N	%
**Professional dance instructor/s**	61	53.0
**Dance movement therapist**	9	7.8
**Exercise specialist/physical education teacher**	9	7.8
**Trained volunteer older adult dance leader**	6	5.2
**Healthcare provider/practitioner**	4	3.5
**Physical therapist/physiotherapist**	2	1.7
**Group exercise instructor/s**	2	1.7
**Undergraduate dance student/s**	2	1.7
**Robot equipped with ‘cognitive dance therapy’**	1	0.9

Note: Some programs were delivered by providers with more than one area of expertise or more than one instructor type

**Table 6:** Dance program attendance rates.

Dance program attendance/adherence	M (%)	SD	Range (%)	N trials
Program frequency/duration				
2 sessions per week	81.0	13.1	56–100	24
3 sessions per week	85.4	8.8	72–100	17
Program 1-3mo duration	87.2	9.3	61–100	30
Program 3-6mo duration	77.8	10.8	60.5–100	11
Program +6mo duration	67.3	9.5	56–78	4
Attendance rates (all trials)	83.1	11.5	56–100	45
All allocated to group dancing (ITT)	73.4	13.6	56–92.5	11
Program completers only	87.1	9.8	71.3–100	12
Pre-determined dose	89.8	11.0	78.1–100	3

Note: Trials which reported minimum attendance only (e.g. all ≥70% attendance) were documented by the lowest value reported (e.g. 70%).

**Table 7:** Percentage of positive tests in each health outcome domain.

Outcome domain	Studies		Tests	Dance > comparator	Common measures
	N	%	N	% + tests^1^	
Physiological/metabolic	28	27.2	174	29.3	BMI, BP, metabolic responses; BMD
Cardio endurance and strength	48	46.6	118	60.2	6MWT; grip strength; HRR; arm-curl; SFT
Physical/motor functioning	80	77.7	495	48.5	TUG; 5XSTS; balance; BBS; gait; SPPB
Physical activity/exercise levels	12	11.7	47	42.6	IPAQ; CHAMPS; accelerometer
Falls and falls risk (composite)	8	7.8	13	15.4	Number of falls; PPA
Falls self-efficacy/balance	6	5.8	10	40.0	MFES; FES-I; ABC
Cognitive and executive function	27	26.2	146	16.4	TMTs; Stroop; DS; LM1&2; RAVLT; Corsi
Everyday cognition (composite)	11	10.7	12	25.0	MMSE; MoCA
Brain health/neurological	5	4.9	132	18.2	Regional volume, diffusivity, connectivity
Mental health/emotional function	26	25.2	47	38.3	GDS; BDI; STAIT; Perceived stress; GSE
Wellbeing and quality of life^2^	25	24.3	52	34.6	SF-12; SF-36; SWLS; WHO-QoL-BREF
Psychosocial/social functioning	7	7.0	11	45.5	Lubben SN; UCLA loneliness; SPS
Activities of daily living	9	8.7	18	16.7	IADL; LL-FDI; Barthel Index
Other^3^	19	18.4	40	-	Sleep; PA/dance self-efficacy; Dual task
**TOTAL (quantitative tests)**	**103**	**100.0**	**1319**	**38.6**	

^1^The percentage tests demonstrating statistically significant positive effects (vs. no effect or statistically significant negative effect for dance > comparator group or baseline tests)

^2^Quality of life measures included in dance health studies typically measured health-related QoL, rather than global QoL

^3^Other outcomes evaluated include sleep (quality, time, sleepiness), frailty, physical activity self-efficacy, dance self-efficacy, exercise self-efficacy, motor-cognitive dual tasking, and exercise enjoyment and experiences

BMI = Body Mass Index; BP = Blood Pressure (systolic and diastolic); Metabolic responses = lipids, cholesterol, triacylglycerols, fasting blood glucose; BMD = Bone Mass Density; 6MWT = 6 Minute Walking Test; HHR = Resting Heart Rate; SFT = Senior Fitness Test (includes measures of cardiovascular endurance, strength, and physical function); TUG = Timed Up and Go; 5XSTS = 5 Times Sit-To-Stand; Balance (multiple tests of static and dynamic balance); BBS = Berg Balance Scale; SPPB = Short Physiological Performance Battery; IPAQ = International Physical Activity Questionnaire; CHAMPS = CHAMPS Physical Activity Questionnaire for Older Adults; PPA = Physiological Performance Assessments; MFES = Modified Falls Efficacy Scale; FES-I = Falls Efficacy Scale International; ABC = Activities-specific Balance Confidence scale; TMTs = Trail Making Tests A and B; Stroop colour-word; DS = Wechsler Digit Span backwards, forwards, sequencing (working memory); LM1&2 = Wechsler Logical Memory 1&2 (episodic memory); RAVLT: Rey Auditory Verbal Learning Test; Corsi block test of visuospatial working memory; MMSE = Mini-Mental State Evaluation; MoCA = Montreal Cognitive Assessment; Regional white and grey matter volume, diffusivity, and network functional connectivity using fMRI; GDS = Geriatric Depression Scale; BDI = Beck Depression Inventory; STAIT = State and Trait Anxiety Inventory Y1&2; GSE = General Self-Efficacy scale; Perceived Stress Scale; SF-36/12 = Health-related Quality of Life Short Form 12 (physical and mental component) or 36; SWLS = Satisfaction With Life Scale; WHO-QoL-BREF = World Health Organisation Quality of Life scale; Lubben Social Network Scale; UCLA perceived loneliness scale; SPS = Social Provisions Scale; IADL = Instrumental Activities of Daily Living; LL-FDI = Late Life Function and Disability Instrument; Barthel Index of Activities of Daily Living

A TIDieR item or program characteristic (e.g., physical intensity, challenges) was considered: 1) *Reported* if reasonably clearly and comprehensively described such that a dance health researcher and/or practitioner, or an expert in an adjacent field (e.g. falls prevention) could understand, and in line with generally accepted standards where applicable (e.g., when reporting adherence); 2) *Partially reported* if incomplete information was provided; and 3) *Not reported* if very limited or no information was provided.

Due to the exploratory nature of this scoping review, we took a lenient approach when considering factors to be *partially reported* to ensure programs with particular features were not under-represented. For example, dance programs with intensity goals monitored program intensity and typically aimed to deliver moderate intensity programs. To avoid under-representation of lighter intensity programs, we used program descriptions and supplementary materials to estimate intensity where possible. Standardisation within and between TIDieR items was required and conditions of reporting for each category were developed.

As this scoping review aimed to document all dance health trials and program reporting to date, a critical appraisal or risk of bias assessment was not appropriate [34] and was not performed.

## Results

**Fig 1:**
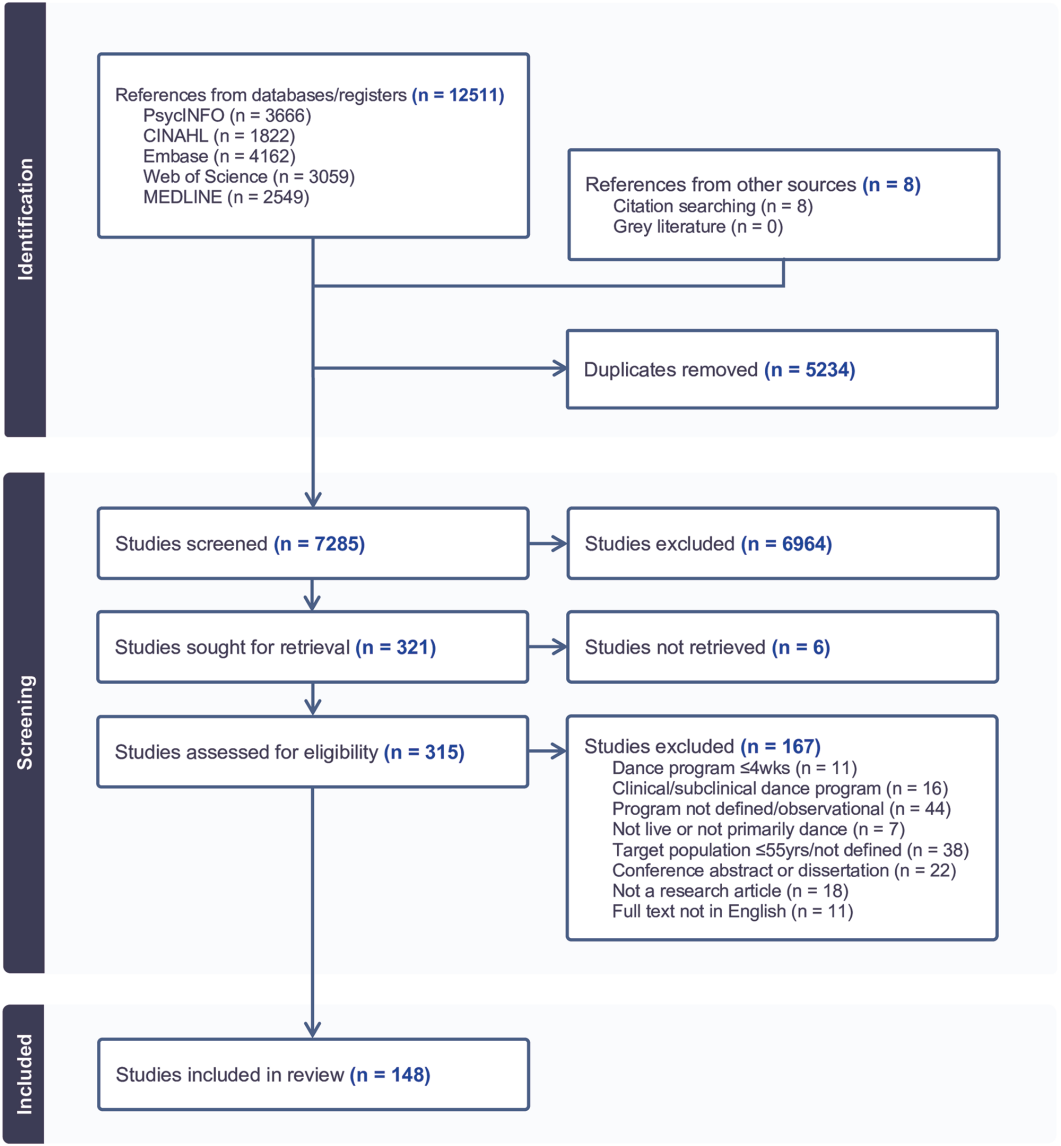
PRISMA-ScR flow chart of the search process and results.

The literature search identified 7285 studies for potential inclusion. Following abstract screening and full-text screening, 148 articles met the scoping review criteria and have been included for analysis (Fig 1).

### Publication and study characteristics

**Fig 2:**
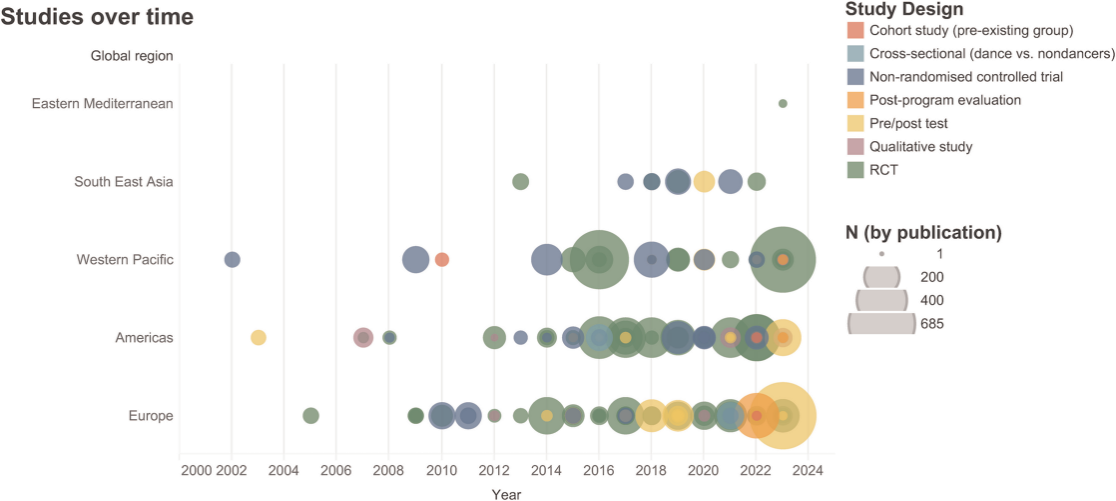
Publications of dance health trials for older adults from 2000 onwards.

The earliest identified dance for health trial was published in 1980 [38]. Publications became more frequent through the mid 2000s, and then increased exponentially, continuing into 2023. Study frequency by global region, study design and sample size from 2000 onwards is displayed in Fig 2 (excluding 4 studies published between 1980–2000; interactive version available at www.dancebrainhub.com).

All 148 publications included in this scoping review are listed in S1 File, and basic descriptors of the trials are presented in S2 File. Most studies were RCTs (75, 50.7%) and non-randomised controlled trials (30, 20.3%), followed by pre-post evaluation trials with no comparison group (21, 14.2%), qualitative studies (13, 8.8%), and a few observational studies of pre-existing or new dance groups (5, 3.4%).

Mixed methods approaches (14, 9.5%) have become more common in the last decade, but rather than synthesising qualitative and quantitative findings, most studies presented results sequentially [39–51]. Of the 26 studies that included qualitative approaches, thematic analysis was the most frequent method of data analysis. A few studies published from 2020 onwards performed process evaluation [39, 40, 44, 52]. Notably, older adult program participants have increasingly been asked to evaluate and provide feedback on program experiences via surveys and interviews. A few recent studies also sought the perspectives of people involved in program delivery including dance program facilitators and support staff [40, 52–54].

### Dance health trial location and setting

The 148 publications included in this scoping review evaluated 115 trials of dance health programs with 8060 older adult participants in total. Sample size of the dance health trials ranged from 5 to 685, with an average 70.4 participants per trial (Median n = 56). Characteristics of the 115 trials in this scoping review including dance program design and delivery are summarised by TIDieR reporting categories, and the dataset is available in the Western Sydney University Research Direct repository [55].

Trial location was documented by World Health Organisation global region [56] and by country. If study country was not reported, the corresponding author’s country was documented. Results presented in Table 1 demonstrate the global reach of dance for health research, with trials conducted across 31 countries.

Trial setting is recorded in Table 2. Most trials were conducted in the community (78, 67.8%) and open to a general population of community-dwelling older people. The exclusion of programs designed for clinical populations limited the number of trials conducted in clinical or higher care settings. Four community-based programs [40, 57–59] switched from delivering in-person dance sessions to live remote at-home dance sessions in response to the COVID pandemic, and a single study was designed for remote delivery [39], all published in 2023 (5 trials, 4.3%). The remaining 110 programs (95.7%) were delivered live, in-person.

### Trial participants

In general, demographic reporting varied widely in quality and consistency. Most dance health trials for older adults were open to adults in late middle-age; 79 trials (68.7%) included adults under 65 years (indicated by minimum age, inclusion criteria, or mean age (years) +/-1.5SD). Average age of participants was reported in 103 trials (89.6%) and ranged between 59 and 86 years, M(SD) 71.1yrs (6.0). Only 50 trials (43.5%) reported a minimum and maximum age. Where reported, the age gap between the youngest and oldest participants in the dance trials ranged from 4 years to 42 years (M(SD) 18.3(8.6)). Thirty-three trials (28.7%) recruited ‘oldest old’ adult participants aged ≥85 years.

No programs were designed for men, but 22 dance programs (19.1%) were designed for women only. Gender was not reported in 9 trials (7.8%), 29 trials (25.22%) recruited female participants only, and 77 (70.0%) were mixed gender groups. Most dance for health study participants were women. Mixed gender trials had 75.0% female participants on average. The highest proportion of female participants in dance programs were in outpatient settings (97.1%), and independent living communities (86.5%), and the lowest proportions of female participants were in aged-care (72.0%) and inpatient (62.3%) settings.

Of the 61 dance programs conducted in culturally diverse countries/regions (United States, Canada, Australia, Western Europe), just 5 programs were devised for people with global majority backgrounds, and 4 of these programs evaluated successive iterations of the BALIMOS^™^ Latin dance program for the Latinx community in Chicago [47, 60–65]. Overall, only 7 trials (6.1%) were offered to participants on low-incomes, and only 2 (1.7%) were conducted in rural or remote areas. Overall, race and ethnicity were so infrequently and inconsistently reported that it was not meaningful to summarise the findings.

Over a third of dance health trials recruited low active (16, 13.9%) or inactive (25, 21.7%) older adults, 4 trials (3.4%) were conducted with active participants only, and the remaining 70 (60.9%) did not consider level of physical activity as recruitment criterion. Trials varied in their mobility-related inclusion criteria, ranging from specifying participants be predominately wheelchair-users, have mobility limitations, to being able to walk independently with walking aids and/or without walking aids only. Other specifiers were ‘healthy’ (6 trials, 5.2%), at risk for disability (2 trials, 1.7%), pre-frail or frail (3 trials, 2.6%), low-functioning (1 trial, 0.9%), post-menopausal (2 trials, 1.7%), and 6 trials (5.2%) with varied criteria related to falls (no falls history, falls history, fear of falling, at risk of falls).

Just 24 trials (20.9%) considered previous dance experience as recruitment criteria. Of those, 11 trials (9.6%) recruited older adults who had not been attending regular dance sessions for a specified period (between 1 and 5 years), 3 trials (2.6%) recruited dance novices only, and 7 trials (6.1%) recruited beginners in a dance style, while 3 further trials (2.6%) recruited older people who were already engaged in regular dance sessions. Only 7 trials (6.1%) reported previous dance experience as a relevant participant characteristic. Dance experience was variously reported as n completed ≥ 5 dancing classes/previously danced; years of practice; n currently dancing; and past dance experience; high, moderate, novice.

### Program properties and characteristics

#### Dance style/genre

The style or genre of each dance program was documented and aggregated into categories presented in Table 3. An extensive range of dance styles are represented in the research literature: 1) Traditional and modern forms of folk, national or social dances; 2) Programs delivering a mix of dance styles, including specialised dance programs for seniors; 3) Formal, structured performance dance styles from the Global South and North; 4) Aerobic exercise dance; 5) Ballroom dance; 6) Creative dance programs with predominately improvised, guided discovery, or creative choreographic tasks; and 7) Dance Movement Therapy (DMT). There were only 3 DMT programs, and these shared many similar properties with the creative dance programs, and so the two categories were integrated.

#### Rationale and development

A clear rationale, theory, or goal for the dance program was *reported* in 78 trials (67.8%), and *partially reported* in 26 trials (22.6%). Outside dance movement therapy (DMT), there were limited theory-informed approaches to program design or delivery. Examples of the few theory-informed programs included: 1) BALIMOS^™^ Latin dance program had social-cognitive theory-informed teaching and discussion sessions with health education and aimed to address outcome expectations, provide social support, and boost self-efficacy in order to increase lifestyle physical activity [60]; 2) GERAS dance was based on motor learning principles and Agility, Balance and co-ordination (ABC) to boost movement efficiency [66]; 3) A Ballroom dancing program considered mobility requirements, pedagogical progression of skill acquisition, and familiarity with the dances, and described maintaining pedagogical flexibility to accommodate the participants’ needs while using scaffolding and peer discussion to provide social support for learning, and boost self-efficacy and skill mastery [44, 53]; and 4) A Tap dance program used the three-stage learning theory (cognitive, associative, and autonomous stages) basic training principles [e.g., overload, progression, reversibility, 67]. Overall, as previously argue by Fortin [19], there was limited description of dance pedagogy and teaching approaches.

The rationales provided for dance programs were wide-ranging and diverse and included: Dance as an activity to promote creative aging, with opportunities for self-expression, artistry and play; dance as a recommended form of exercise for older people–particularly people who do not like exercise—low-impact, with motor-skill learning and cognitive challenges and requiring skill acquisition; dance for falls prevention; the therapeutic properties of dance used for relaxation and stress reduction; population-specific cultural relevance of dance styles and traditions; dance as a fun, popular, low-cost, scalable, inclusive, accessible, modifiable, and sustainable activity that tends to have high adherence rates and can be delivered in a wide range of settings; the low barrier to entry allows beginners to participate; programs can be delivered remotely and participants can dance safely at home; dance as a meaningful experience shared with a group, improving social connectedness, and tackling loneliness and social isolation.

Only 25 studies (21.7%) clearly described the dance program development processes. Six studies (5.2%) assessed programs that had been piloted prior to the trial [27, 40, 47, 68–72], and 12 (10.4%) examined pre-existing programs, some with adaptations for non-clinical older dancers including Healthy-Steps [70, 73], dance movement therapy (DMT) [74], and SeniorDance [75]. The remaining 97 trials (84.3%) evaluated new programs. Seven programs (6.1%) were designed with input from health professionals, including geriatricians, physical therapists, and exercise scientists [e.g., 46, 59, 66, 76], while most studies did not define the expertise of the research team. Several of the studies included in this review designed programs that were piloted and evaluated, revised and scaled up, and then evaluated again, including BALIMOS^™^ [27, 40, 46, 47, 77], GERAS dance [66, 77], and a U.K-based community program [42, 46].

Remarkably, only 7 trials (6.1%) involved older adults in dance program development. Participant input ranged from music choices [78, 79], focus groups conducted to examine attitudes to dance and exercise and guide program design [47], to co-design with an independent panel of community-dwelling older adults (no further details) [42, 46]. One Australian-based ballroom dance program piloted a dance program for 12 weeks and modified the program in response to participant feedback on dance content, music, teaching methods, and movement suitability and comfort, and involved a second group of older adults in selecting appropriate dance instructors [27]. Substantive participant input into dance for health study or program design through community consultation or co-design remains rare.

#### Program structure and approach

Fortin defines content-centred dance programs as delivering predefined dance sessions with distinct stages and structured dance exercises of either choreographed movement or creative tasks and often focus on developing technical dance skills [19]. Conversely, participant-centred programs are flexible and dynamically responsive to a group in the moment and tend to focus on free expression, exploration, and guided discovery [19]. Most of the dance programs (93 trials, 80.9%) were content-centred and structured, but 5 (4.3%) of the creative dance or dance movement therapy programs were participant-centred, and 12 trials (10.4%) combined content-centred and participant-centred approaches [e.g., 40, 43, 46, 48, 54, 59, 80–82]. The remaining 5 trials (4.3%) were inadequately described.

Both content-centred and participant-centred programs maintained a familiar structure that was common across most programs included in this review. Dance sessions started with a warm-up, followed by a main section to learn and perform set dance sequences and/or engage in improvisation and creative tasks, and ended with cool-down, relaxation and/or stretching exercises. The proportion of class dedicated to each section varied but was typically a warm-up of 10–20 minutes, 30–40 minutes training, and a 10 minutes cool-down. Programs with atypical structures include the Dancing Heart program with 30-minutes dancing and 30-minutes storytelling and reminiscing [48], programs with creative workshops to generate a performance piece [43, 83], and programs with partnered dance styles such as Tango, Ballroom, and Latin which finished with a period of ‘free practice’ or held dance parties where leaders chose the upcoming moves out of a set of possible combinations introduced during the sessions [e.g., 47, 71, 84, 85].

#### Physical intensity

The physical intensity of the dance program was *reported* in 32 trials (27.8%), and *partially reported* in 17 trials (14.8%). Estimated or established dance program intensity for these trials varied considerably across programs. Overall, for dance health trials with intensity indicators, 12 programs (24.5%) were light intensity, 10 programs (20.4%) were light-moderate intensity, 27 programs (55.1%) were moderate or moderate-vigorous intensity.

Intensity monitoring also varied and reporting was again inconsistent. Trials that monitored intensity mostly used cardiac monitoring (devices or self-administered) and/or rates of perceived exertion (RPE), but a few trials used accelerometers. Some programs had individualised heart rate targets while others had group-level/general target ranges. The timing of monitoring (i.e. when and how many times during the dance session and how many sessions total) varied widely.

Trials that monitored intensity at several points during a dance session described intensity levels varying. Rodrigues-Krause [28, 86] rigorously tested physical intensity across each section of a mixed styles dance session for older adults. The study reported that the warm-up was ~55% VO2peak, a section with traveling steps was ~62% VO2peak followed by choreography learning at ~63% VO2peak, and then a period of continuous repetition of learned choreography at ~69% VO2peak, with overall intensity at approximately 60% VO2peak.

#### Challenges and opportunities

This scoping review identified four challenges and opportunities that were regularly described features of dance health programs for older people hypothesised to impact health and wellbeing: 1) Balance challenges; 2) Motor-skill learning challenges; 3) Cognitive challenges; and 4) Creative and artistic opportunities. The challenge(s) presented by the dance program in one or more of those four specified domains was *reported* in 36 trials (31.3%), and *partially reported* in 20 trials (17.4%), including trials that specified low level challenges (e.g., ‘dances were simple and easy to learn’ would indicate low motor-skill learning challenge). Many dance trials described their programs as challenging a particular area of functioning but did not explain how. A total 59 trials (51.3%) provided insufficient information to establish the level of any of the above challenges and opportunities, and which therefore either did not feature in the programs or did feature but were *not reported*.

The types, quantity, and level of the challenges varied across programs. Challenges to balance were described in 19 dance trials (16.5%) and included multidirectional steps, heel raises, single-leg stances, and pivots or turns. Motor-skill learning challenges were described in 33 trials (28.7%) and included increased difficulty of individual dance moves and sequences of movements, faster rhythms, and layered dance components (e.g. footwork combined with arm patterns) to challenge co-ordination.

Twenty-six trials (22.6%) reported designing cognitively demanding dance programs and included the learning, memorisation, and immediate and delayed recall of dance sequences, dances, and series of dances, learning and switching between leader and follower roles in partnered dances, and dual tasks such as singing while dancing. Finally, creative and artistic opportunities were described in 27 trials (23.5%) and included emphasising the artistry of a style of dancing, creative and improvisational movement tasks, guided discovery improvisation using imagery, and ‘free practice’ in partnered dancing. Remarkably, the remaining 88 dance trials (76.5%) made no mention of opportunities for creativity or artistic expression at all.

#### Standardisation and tailoring

The degree of standardisation and tailoring (adaptation or personalisation) of a dance program was *reported* in 42 trials (36.5%), and *partially reported* in 11 trials (9.6%). Nineteen programs (16.5%) were standardised, including programs that were manualised with no tailoring reported [e.g., 27, 47, 87, 88], standardised with inbuilt difficulty levels (e.g., seated, standing with support, or standing without support options) [e.g., 50, 73], and pre-existing programs adapted to older adults, but not personalised, [e.g., 66, 71, 89, 90]. Thirty-three (28.7%) were tailored programs most commonly adapted or personalised to facilitate participation and accommodate the needs and capabilities of the group [e.g., 44, 67, 68, 91, 92], and occasionally in response to participants’ interests and preferences [e.g., 42, 53, 93].

#### Dose

Dose of group dancing was reported by session length (minutes), session frequency (per week), program duration (weeks), and total dose (hours) for all trials except studies of pre-existing or ongoing dance programs or groups with no set duration. The dose of dancing offered in the trials varied substantially and is presented in Table 4. Trials typically offered 60 minutes of group dancing, 2–3 times per week, for 12–16 weeks, but the programs ranged from a single 15-minute session, once per week for 6 weeks (1.5 hours total) through to 45-minute sessions, 4 times per week for 18 months (234 hours total). Seven trials (6.1%) included movement tasks to complete alone at home [50, 66, 77, 91, 94–96] to increase dose.

#### Program facilitators

Dance program facilitator expertise was *reported* in 85 trials (73.9%), and *partially reported* in 5 trials (4.3%), and is recorded in Table 5. Notably, very few studies reported the prior dance program delivery experience of providers, and only 16 trials (13.9%) provided any training for program facilitators.

Thirty-four programs (29.6%) were delivered by a single dance instructor, and 35 programs (30.4%) had more than one provider (40% did not report). For programs with more than one facilitator, 6 (5.2%) were delivered using a co-teaching model, and the remaining 29 (25.2%) were delivered with assistance or supervision by providers with a range of backgrounds including musicians, healthcare providers or practitioners, trained volunteers, professional dancers, dance students, and carers or family. A further 6 programs (5.2%) were delivered by trained volunteer dance leaders using a ‘train the trainer’ model, including 4 programs (all BALIMOS^™^) which switched program delivery after 4 months to trained volunteers recruited from older adult program participants [60].

Class sizes were reported or estimated for 59 trials (51.3%) and ranged from 4 to 41 participants (M = 19.1, Median = 18), with no consistent pattern across dance styles or contexts (e.g., aged care dance programs reported class sizes of 6–8, 12, 10–15, 15, 10–20, and 25). Participant to provider (student-teacher) ratios ranged from 1.5 to 41 (M = 13.2, Median = 11).

#### Facilities and infrastructure

Only 26 trials (22.6%) *reported*, and 21 trials (18.3%) *partially reported* the types of facilities and infrastructure necessary for delivering the dance program, including room size requirements, type of flooring, and room and venue accessibility. Community-based programs were delivered in community centres including venues for seniors only, fitness or recreation centres, religious venues, local health centres and rehabilitation units, performing arts venues, local community dance studios or dance halls, professional dance company studios, and university sports or dance facilities.

In general, little infrastructure or equipment was necessary for in-person program delivery. Twenty-two trials (19.1%) used chairs (sturdy and without arms) to deliver seated and supported standing dance exercises, 2 trials (1.7%) used a ballet barre for support and 1 aged-care trial (0.9%) had participants dance in their wheelchairs. Only 3 trials (2.6%) used headset microphones and a sound system with adjustable volume to accommodate older adults with hearing difficulties [44, 66, 77]. A further 3 trials (2.6%) had sound systems with adjustable beats per minute (bpm; which can now be achieved using a free music app). Several trials used costumes, props such as streamers, tissue paper, or hats, percussion instruments, and/or strength and conditioning tools like tennis balls and stretchy bands.

The 5 trials (4.3%) delivering live at-home dance sessions required a screen device with videoconferencing capabilities and an internet connection. One of those trials provided all the necessary technology resources [39], while the rest relied on participants’ own resources [40, 57–59]. Two of the at-home trials described providing technical support before and during the dance sessions and instructed participants on how configure their home dance space to ensure safe conditions [40, 59].

### Outcomes

#### Program adherence

Dance program adherence was documented for trial participants allocated to group dancing and *reported* in 48 trials (41.7%), *partially reported* in 4 trials (3.5%), and not applicable to 8 observational trials (7.0%) of ongoing programs or dance groups. In general, reporting lacked clarity. Authors more often reported study retention or attrition rates (which are out of scope for the current review).

Only 11 trials (9.6%) reported attendance rates for all participants allocated to group dancing (intention to treat (ITT)), 16 trials (13.9%) reported attendance for program completers only, and 5 trials (4.3%) reported rates for participants who had reached a pre-determined attendance threshold (e.g. ≥70% or 20 sessions, with underdosed participants typically excluded from the denominator). The remaining 21 trials (18.3%) did not specify how attendance was calculated. Dance program attendance rates are documented in Table 6.

**Fig 3:**
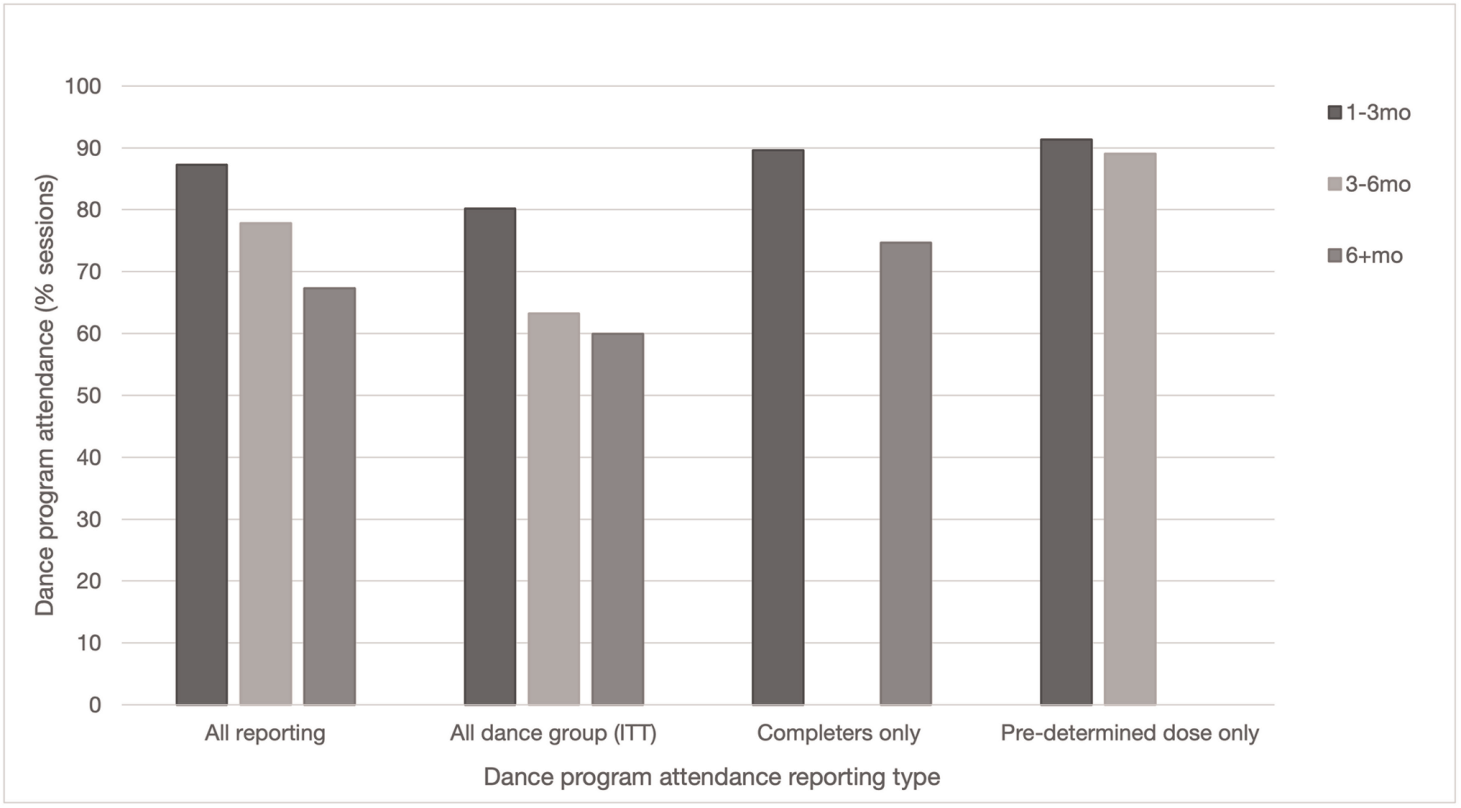
Dance program attendance at 1-3mo, 3-6mo and 6+mo by attendance rate reporting types.

Poor reporting inflated attendance rates. While rates of attendance were similar for programs with 2 or 3 sessions per week, attendance was lower with longer programs. Programs lasting 1–3 months reported average attendance rates of 87.2% sessions, 3–6 month programs had 77.8% attendance, and programs over 6 months reported 67.3% attendance. Importantly, there was markedly lower attendance and more pronounced decreases in trials using rigorous ITT reporting (Fig 3). Attendance varied somewhat across settings, but there were insufficient data to make effective comparisons. General reasons for non-attendance included transport issues, poor weather, health issues, visits from family, and holidays. Two trials (1.7%) for older people living in low socio-economic areas (low-SES) cited additional barriers of work or work status change, caregiving responsibilities (spouses and grandchildren), and not being able to afford or organise alternative transport [65, 78].

While a few trials with intensity goals reported adherence to the physical intensity of dance activities, few further adherence-related factors were considered (e.g., time dancing vs. at rest, engagement with activities). One trial (0.9%) used the Arts Observational Scale [97] to construct observer reports (mixed methods) of positive mood, levels of distraction, post-program relaxation, creative expression, and social interactions among older adult participants of a creative dance program delivered in an inpatient setting [98]. The trial reported participants were physically and mentally engaged with the program, willing to be creatively expressive, and were distracted from their health issues and surroundings.

#### Program safety

Program safety was only *reported* for 28 trials (24.3%). Two trials (1.7%) were described as ‘safe’, 15 (13.0%) reported no adverse events, 3 (2.6%) reported no serious adverse events, and 2 (1.7%) reported no injuries. Six trials (5.2%) reported a single adverse event. Of these, 3 (2.6%) reported injuries (2 knee injuries), 2 (1.7%) reported dancing exacerbated prior injuries or joint problems, and 1 (0.9%) reported a fall (caused by loose shoelaces) which did not result in injury. Dance can be considered a safe activity in later life, and authors indicated that older adults should expect light muscle soreness in response to dancing.

#### Outcome domains and program success

Research trials (103, 89.6% of total trials included in the review) evaluated the health and wellbeing benefits of group dancing across a wide range of outcomes. Identified health and social outcome domains with frequency counts are documented in Table 7 using the percentage of statistically significant positive tests (dance > comparator group or baseline tests) out of all tests conducted in each outcome domain. Table 7 further reports the quantitative measures and tools that appeared most frequently to assess change across the dance health trials. Most measures and tools included in the studies are well-evidenced, standardised, valid, and reliable, and appear frequently in health-related research with older people.

Physical and motor functioning was the most prominent outcome domain evaluated for improvements across 80 trials (77.7% of trials including quantitative measures). Studies most frequently measures of static and dynamic balance, mobility and agility, gait, flexibility, and functional reach. Physical and motor functioning demonstrated statistically significant improvements after group dancing in 48.5% of 495 total tests conducted, one of the more reliable outcome domains.

Cardiovascular endurance and strength was the second most prominent outcome domain evaluated in 48 trials (46.6%). Measures most commonly assessed cardiovascular fitness (walking and stepping endurance, VO2 capacity), grip strength, and lower and upper limb strength. Cardiovascular endurance and strength showed improvements after group dancing in 60.2% of 108 total tests conducted and was the outcome domain that showed the most consistent and reliable positive response to dancing.

Next were physiological and metabolic outcomes evaluated in 28 trials (27.2%). Measures most frequently assessed body composition, blood pressure, blood biochemistry (lipid, glycaemic, and cholesterol), and bone mineral density; 29.3% of the 174 total tests conducted showed improvements.

Cognitive and executive functioning were the fourth most prominently reported outcomes in 27 trials (26.2%). Studies predominately included measures assessing the executive function domains of cognitive flexibility/task switching, response inhibition, verbal, non-verbal, and visuospatial working memory, as well as verbal, non-verbal, and spatial memory and learning, episodic memory, and processing speed. Cognitive and executive functioning showed poor response to group dancing; just 16.4% of the 146 total tests conducted showed positive benefits.

In related domains, everyday cognition was evaluated in 11 trials (10.7%) using composite measures and had marginally more reliable improvements in response to dancing; 25.0% of 12 tests were positive. The 5 trials (4.9%) assessing brain structure and function using neurological measures, typically regional white and grey matter volume, diffusivity, and network functional connectivity using fMRI, reported results of 132 individual tests. However, only 18.2% of test outcomes showed evidence for reliable changes in neural structure or function.

Mental health and emotional functioning was evaluated in 26 trials (25.2%), and most frequently included self-report measures of depression, anxiety, stress, general self-efficacy, and morale, and showed improvement in response to dancing in 38.3% of the 47 tests that were conducted. Similarly, wellbeing and quality of life (QoL) measures were included in 25 trials (24.3%), predominately self-reported health-related QoL and life satisfaction. Studies reported increased wellbeing and QoL in 34.6% of the 52 total tests conducted. Psychosocial and social functioning was evaluated in 7 trials (7.0%), and included measures of social network, perceived loneliness and social support, and demonstrated benefits in response to dancing in 45.5% of the 11 tests conducted.

Benefits to falls self-efficacy and balance confidence were assessed in 6 trials (5.8%) and also showed a relatively reasonable rate of improvement; 40.0% of the 10 tests conducted were positive. However, falls and falls risk (composite measures) were evaluated in 8 trials (7.8%) and demonstrated limited response to dancing; only 15.4% of the 13 tests conducted showed positive change. Finally, activities of daily living were assessed for improvements in 9 trials (8.7%) and showed positive improvements after group dancing in only 16.7% of 18 tests conducted. Other health and social outcomes evaluated in the dance for health trials included sleep (quality, time, sleepiness), frailty, physical activity or exercise self-efficacy, dance self-efficacy, motor-cognitive dual tasking, and exercise enjoyment and experiences.

#### Outcomes by dance style

Table 8 presents the percentage of positive tests across health and wellbeing outcome domains for each style of group dancing. There was substantial variability in the proportion of positive test outcomes achieved across different dance styles. Overall, ballroom dance achieved the most consistently positive results across all domains, with twice the success rate of folk/social/national dance and creative or dance movement therapy (DMT) programs and also achieved better cardiovascular endurance and strength and physical functioning outcomes than aerobic/exercise dance. Global performance styles were also associated with strong physical health benefits, but fewer positive cognitive and mental health outcomes.

**Table 8:** The percentage of statistically significant positive test outcomes and total number of tests conducted in each health and wellbeing outcome domain across dance style/genres for all trials.

Outcome domain	Studies	Folk/social/		Mixed/		Global		Creative/		Aerobic		Ballroom		TOTAL	
		national		specialised		performance		DMT		dance		dance			
		(n = 28)		(n = 27)		(n = 24)		(n = 13)		(n = 12)		(n = 10)		N = 114	
	N	%+	(n)	%+	(n)	%+	(n)	%+	(n)	%+	(n)	%+	(n)	%+	(N)
Physiological/metabolic	28	21.4	(28)	34.1	(82)	6.3	(16)	71.4	(7)	25.0	(40)	100.0	(1)	29.3	(174)
Cardio endurance and strength	48	42.4	(33)	56.3	(16)	50.0	(12)	25.0	(8)	74.1	(27)	90.9	(22)	60.2	(118)
Physical/motor function	80	35.8	(176)	55.3	(94)	59.6	(104)	34.0	(47)	55.6	(36)	71.1	(38)	48.5	(495)
Physical activity/exercise	12	38.5	(26)	25.0	(4)	69.2	(13)	-	-	0.0	(4)	-	-	42.6	(47)
Falls and falls risk (composite)	8	33.3	(3)	0.0	(7)	-	-	-	-	-	-	33.3	(3)	15.4	(13)
Falls self-efficacy	6	-		40.0	(5)	40.0	(5)			-	-			40.0	(10)
Cognitive & executive function	27	19.5	(41)	19.0	(58)	0.0	(7)	20.0	(5)	9.1	(22)	15.4	(13)	16.4	(146)
Everyday cognition (composite)	11	0.0	(2)	60.0	(5)	0.0	(1)	0.0	(2)	0.0	(1)	0.0	(1)	25.0	(12)
Brain health/neurological	5	8.1	(62)	27.1	(70)	-	-	-	-	-	-	-	-	18.2	(132)
Mental & emotional function	26	50.0	(10)	50.0	(12)	14.3	(7)	44.4	(9)	12.5	(8)	100.0	(1)	38.3	(47)
Wellbeing & quality of life	25	25.0	(20)	50.0	(12)	28.6	(7)	25.0	(4)	40.0	(5)	50.0	(4)	34.6	(52)
Psychosocial/social function	7	50.0	(4)	100.0	(2)	0.0	(1)	0.0	(1)	-	-	33.3	(3)	45.5	(11)
Activities of daily living	9	0.0	(9)	50.0	(4)	0.0	(1)	0.0	(1)	0.0	(1)	50.0	(2)	16.7	(18)
Other^1^	19	82.6	(23)	50.0	(4)	80.0	(5)	0.0	(5)	0.0	(1)	50.0	(2)	-	(40)
**TOTAL % POSITIVE (N)**	**107**	**31.4**	**(439)**	**37.9**	**(377)**	**48.3**	**(180)**	**32.2**	**(90)**	**38.5**	**(143)**	**63.3**	**(90)**	**38.6**	**(1319)**

^1^Other outcomes evaluated include sleep (quality, time, sleepiness), frailty, physical activity self-efficacy, dance self-efficacy, exercise self-efficacy, motor-cognitive dual tasking, and exercise enjoyment and experiences.

Trials delivering mixed style and specialist programs demonstrated the most reliable benefits to everyday cognition and brain health, but middling benefits to cardiovascular endurance and strength and physical functioning, and no reduction in falls or falls risk, but improved falls self-efficacy. Along with ballroom and mixed and specialist programs, folk/national/social and creative dance/DMT produced the most consistent benefits to mental health and emotional wellbeing, but poorer physical health outcomes.

#### Comparator

There was more than one comparator in 12 trials (10.4%) for a total of 130 comparator groups. Dance was most frequently compared to usual care/daily living (55 groups, 42.3% of total groups), and 5 of these groups were wait-listed to later receive the dance program. For the 55 comparator groups (42.3%), allocated an activity, dance was most commonly compared to exercise (34, 26.9%). including walking (7 groups, 5.4%), aerobic training with strength and conditioning (6 groups, 4.6%), falls prevention exercise and balance training (6 groups), Tai Chi (3 groups, 2.3%), strength and conditioning (3 groups), stretching (2 groups, 1.5%), as well as swimming (1 group, 0.8%), running (1 group), or Pilates (1 group).

While the comparator exercise activities varied widely in physical intensity, many (but not all [99]) were intensity matched to the dance programs [e.g., 69, 84, 87, 100, 101]. However, while some programs monitored the intensity of both dance and exercise activities using processes such as regular HR monitoring, other programs simply stated that the activities were equivalent intensities and provided no further information.

Group dance was sometimes compared to other social, educational, or arts activities (11 groups, 8.5%), including health education (6 groups, 4.6%), interactive social or discussion groups (3 groups, 2.3%), learning a musical instrument (1 group, 0.8%), and non-group dance (1 group). Notably, for comparator groups that were allocated an activity (where the activity was adequately described), most were group activities, controlling for some of the social opportunities associated with group dancing.

#### Quality of program/intervention reporting

**Fig 4:**
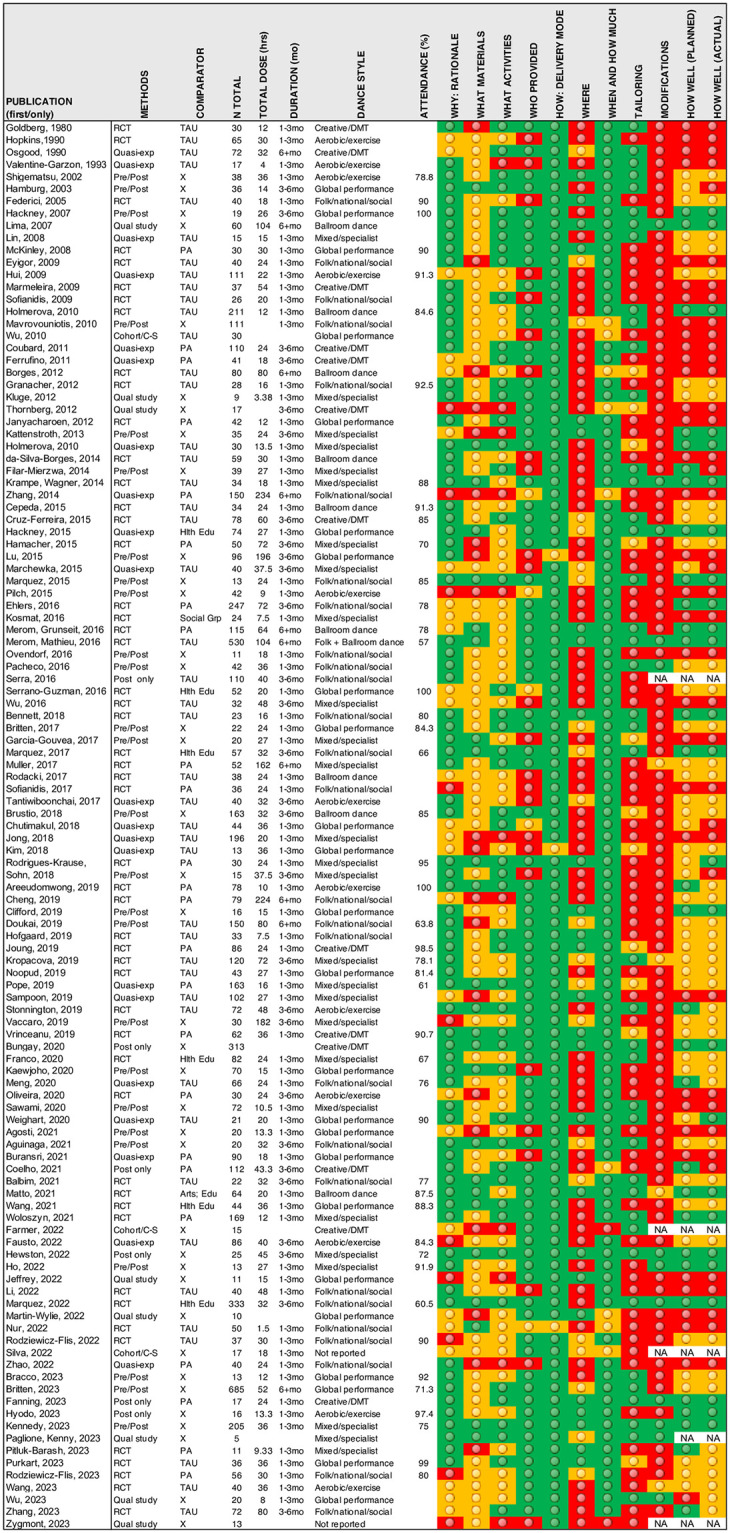
Dance health trial study design and TIDieR intervention reporting evidence map. Reporting quality: Red = Not reported; Amber = Partially reported; Green = Reported.

An evidence heat map indicating the quality of dance heath trial reporting for each TIDieR category is presented in Fig 4. Descriptions of WHERE (locations and necessary infrastructure), TAILORING (program personalisation or adaptations), program MODIFICATIONS, and HOW WELL (intervention delivery monitoring and implementation assessments including adherence and fidelity) were under-reported. Descriptions of WHAT MATERIALS (informational and physical) and WHAT ACTIVITIES and/or processes were also insufficient to understand how dance programs varied in content, structure, and key components.

## Discussion

This scoping review of 148 research publications found global interest in the potential of group dancing programs to support health and wellbeing in later life, with substantial increases in study frequency since 2010. The review established variability in the properties and characteristics of the group dance programs for older adults represented in the research literature including a variety of dance styles, program structures, dance content, physical intensity, challenges, creative opportunities, standardisation and tailoring, program facilitation, and dosage. The benefits of dancing were evaluated across a comprehensive range of health and functional domains, indicating the prospective broad utility of dance as a health resource. However, program success varied across health domains and dance styles. Reporting of both participant characteristics and interventions was inconsistent and often inadequate.

### Evidence gaps and opportunities

#### Gap 1: Participant demographics

The first evidence gap identified in this review concerns the demographic characteristics of participants. Poor demographic reporting made it impossible to summarise beyond age and gender, undermining our ability to understand who is represented by this research, who may benefit most from dance activities, and what barriers to participation may exist for particular groups. Although older age is typically defined as ≥65 years, a large majority of these research trials for ‘older adults’ included participants in late middle-age, and excluded studies had adults in their 40s and early 50s. Conversely, a third of trials recruited participants in advanced older age (≥85 years), including 14 community-based programs. The impact of catering to such a broad age range of participants on dance program implementation and efficacy is undetermined and warrants further investigation.

Most trials offered mixed gender dance programs but recruited more women (4:1 female to male) than estimates for recreational dance participation in Australia and the U.K. (approximately 2.5:1 [25, 102]). Older men’s participation was more prevalent in aged-care and inpatient settings, and in partnered dance, creative or mixed style programs. Older adult men who are ‘captive audiences’ and have ready access to dancing opportunities (and perhaps peer support or modelling) may be more willing participants. Despite research indicating no gender differences in dance confidence or dance self-efficacy among older people [103, 104], the work covered in this review suggest that recruiting men into community dance remains challenging.

The global reach of the studies indicated some racial diversity in dance for health research participation, especially in Latin America and Asia, but our search yielded no published papers detailing trials from Africa or the Caribbean. Of the dance programs conducted in culturally diverse countries/regions, only two U.S.-based trials were specifically devised for older people with global majority backgrounds [105, 106]. Additionally, our search revealed a deficit of dance programs directed toward older people on low incomes or living in low-SES areas, LGBTQI+ individuals, indigenous peoples, people in rural and remote areas, and people experiencing loneliness and social isolation. These groups face greater health disparities [107], reduced access to arts opportunities [2, 3, 108–111], and more significant barriers to physical activity participation [112–115]. From our scoping review, we can conclude that group dance remains under-utilised as a health and social resource for these populations.

#### Gap 2: Program attendance and acceptability

Authors of primary studies relied on strong attendance to maintain that dance is a universally fun, acceptable, and appealing physical activity for older people. However, although average program attendance was 83.1%, across the studies included in this scoping review, reporting was regularly misleading, and adherence has likely been overstated. For example, one study reported 92% attendance, but excluded 26% of the cohort for completing less than half the sessions [82]. Another reported a “high and consistent” [p12] attendance rate of ~70% over 12 months, but lost 82.9% of participants by study completion, who were excluded from the attendance estimate [42].

For trials longer than three months reporting data for all participants, attendance was ~60%, comparable to adherence to other community-based exercise programs for older adults such as aerobic and strength training, group walking, yoga, and at-home falls prevention training [115–117]. Limited analysis of differential attrition was evident, with only one trial reporting selective attrition based on poorer cognitive abilities and among men [87]. Given the above findings, we cannot assume that dance is an acceptable activity for all older people and should acknowledge that group dance participation is likely to require similar supports for uptake and maintenance as other exercise activities and similar attention to personal factors affecting participation.

#### Gap 3: Theory-driven research and program-specific rationales

Rationales for providing dance to support the health of older adults varied widely, emphasising the potential benefits to a range of health outcomes through multiple mechanisms and across different contexts. However, the lack of program-led rationales and theory-informed program design limits our ability to map and explain the current research and develop testable hypotheses [16, 19, 32]. Many program rationales were based on unverified assumptions regarding the dance program acceptability. For example, dance was regularly described as a fun activity, yet only 12 trials (10.4%) actually surveyed or confirmed through focus groups that older people (mostly) enjoyed participating.

Factors determining dance activity selection and design were often unclear; whether the choice of activity types—style, structure, content, pedagogy, delivery, and even dose—was primarily informed by previous research or theories of change, or external factors such as availability, funding, and facilitator experience [19, 32]. While some studies tested assertions regarding the properties of dance programs identified as driving health outcomes (e.g., introducing progressive cognitive challenges to dance programs to improve cognition versus intensity-matched group exercise [101, 118]), many did not.

Few dance programs were theory-informed, with limited consideration of conceptual models or frameworks to test hypothesised mechanisms underlying health and behaviour change. Relevant frameworks include experimental medicine frameworks such as therapy and resilience [119], motor-skill learning principles [120, 121], social cognitive theory [122–124], self-determination theory [123, 125, 126], and models of health behavioural support [114, 123]. Given the prevalence of these approaches in the broader health intervention literature for older adults (e.g. physical activity, health and behaviour change [5, 114, 127, 128]), their limited application in dance health research was unexpected. A clear conceptual framework referencing well-evidenced theories should underpin program development and study design to understand how interventions cause change and identify key program properties that reliably support activity participation and generate health benefits as well as factors that moderate these processes [21, 24, 32, 119, 129].

#### Gap 4: Participant and practitioner perspectives

The fourth critical gap in the dance health literature concerns the lack of input, experiences and perspectives of older adults and people involved in dance program delivery. Only one dance program was co-designed with older people [46], and community consultation was scarce. Incorporating participant and artist voices in arts health is essential for successful interdisciplinary learning and research collaboration to optimise health and social impacts while appreciating different ways of knowing, valuing, and investigating arts and cultural experiences [19, 33].

Greater attention to participant and practitioner perspectives and experiences can identify factors that influence the uptake, adherence and engagement with dancing, similar to other physical activities [115, 124, 126, 130]. For example, only two trials described older adults’ motivations to dance [47, 53], and two trials considered dance self-efficacy [27, 87, 104]. There was a general lack of focus on outcome expectations, perceived barriers, preferences for style, content, teaching approaches, intensity, and movement suitability and comfort, as well as program delivery factors such as session timing and frequency, and appropriate instructors. While several studies established the cultural salience of participating in Latin dance for older people with Latinx heritage [71, 105], there is little research exploring the forms of dance older people from other backgrounds consider culturally relevant. Overall, the work included in the scoping review suggests that the research community has minimally engaged with older adults to better understand their motivations, preferences, values, and ideas for dance opportunities.

#### Gap 5: Program suitability

This review has established that dance programs are generally physically safe for older adults, including online programs delivered virtually [131]. However, significant gaps remain regarding program suitability for older participants with distinct needs and capabilities for dancing. The average age gap within programs was 18 years, and the largest gap was 42 years. Ageing affects physical activity capacity, functional mobility, intensity tolerance, and neurocognitive abilities [4, 132–134], all of which impact capacity for participation in complex motor activities such as dance [135, 136]. Dance is a complex social activity that involves motor-skill acquisition with varied task demands, creating many potential impediments to success, particularly in older populations with heterogenic physical and cognitive abilities. Personal factors such as exercise behaviour, prior dance experience, preferences, and mental health are also likely to influence capability for group dancing [124, 137–139].

Accommodating such diversity is difficult. For instance, although hearing impairments are common in older age, only three programs catered to participants with hearing difficulties [44, 66, 77]. Few trials described differences in participant dance capabilities, though three noted heterogeneity in dance learning and progression [27, 44, 87]. While 45 studies (39.1%) recruited either low or high active participants, and 24 (20.9%) considered prior dance experience during recruitment, most trials included participants with varying levels of physical fitness and dance skills. Such disparity in capabilities for dancing may complicate program delivery, affect plans for program progression, and impact participant satisfaction and self-efficacy for dancing [104], and health outcomes.

Most trials reviewed delivered one-size-fits-all dance programs, with only a small proportion tailored and to varying degrees. In contrast, dance programs for younger people are typically available at developmental and skill-appropriate levels. Programs for older adults in the community may be more commonly delivered at different ability levels, or older people may self-select into programs according to their abilities, potentially improving program fit and efficacy. A recent realist process evaluation describes RIPE, an established community dance program provided at various ability levels, which highlighted the importance of ensuring sessions were safe and appropriate, with individuated modifications of dance movements [6]. Furthermore, very few studies assessed differences in benefits associated with dance participation for, for example, older people with poorer health, fitness, physical and cognitive functional performance at baseline, or who have fewer arts or dance experiences compared to their peers.

Another consideration for program suitability is the expertise required to facilitate dance for older adults. As arts approaches in health gain influence, there has been increasing awareness of the skills, knowledge, experience, and training required to deliver arts programs successfully and sensitively for people with diverse needs [19, 140]. Facilitators need to work with people with different capabilities and motivations [44]; understand health issues and orthopaedic limitations to provide suitable challenges and avoid physiological overload [28, 141–143]; modify movements as needed [6, 68, 78]; and create a non-judgemental environment that promotes confidence and a sense of belonging [6, 80, 98, 140]. However, few studies reported previous experience in delivering dance for older people or described the skillsets required. The issue is further complicated by a lack of formal training opportunities or recognised qualifications for dance artists or dance educators who work in a health context (outside dance therapy).

Program-related factors affecting suitability include class sizes and student-teacher-ratios, and the support required from volunteers or supervisors which varied widely between studies and contexts. Only one trial addressed these factors and recommended a teacher-to-student ratio lower than 13 due to participants struggling to hear, follow, and receive sufficient individualised support from instructors ([44]; mean ratio across all programs was 13.2, and ranged 1.5 to 41). Furthermore, a limited number of trials offered program-specific training to providers. Ensuring that programs are well-matched to the capabilities and needs of participants can enhance engagement, improve outcomes, and support the overall effectiveness of dance as a health-promoting activity for older adults [5, 6, 135].

#### Gap 6: ‘Active ingredients’ and health outcomes

The final evidence gap identified concerns potential ‘active ingredients’ of dance programs and their impact on health outcomes. This review identified a broad collection of dance styles and approaches, showing significant variation in factors such as balance, cognitive, and motor-skill learning challenges, physical intensity, and creative opportunities; all likely to drive health outcomes [4, 7, 26, 29, 144–147]. Many of these factors require progressive and individuated challenges to generate health benefits and are often dose dependent [4, 11, 26, 148, 149]. However, the extent to which programs provided sufficient and appropriate challenges across the range of participant capabilities is undetermined. Some challenges may be particularly relevant for content-centred programs with specific motor-skill learning objectives, which constituted the majority of programs in this review, but are less important for participant-centred programs focused on individual movement exploration and personal expression [46, 98, 150].

Dance was most frequently investigated for benefits to physical health, followed by cognitive brain health indicating a primary focus on dance as a multicomponent physical activity for older adults [5, 144, 146–148]. Program success varied substantially across different health outcome categories. Overall, improvements to physical health including cardiovascular endurance and strength, and motor functioning, were more consistent than improvements to cognition and brain health. Benefits to mental health, quality of life, and social functioning, as well as falls self-efficacy were moderate. Across all studies, fewer than 40% of health and wellbeing assessments demonstrated significant positive change in response to dancing.

Most programs were probably under-dosed to improve cognitive function which meta-analyses suggest requires approximately 52hrs total training regardless of intensity [148] or a minimum 175mins per week at moderate intensity and excluding warm-up or cool-down [151]. Achieving an optimal balance between physical and cognitive training may enhance neuroplasticity and processes impacting brain health [152]. Indeed, some content-centred programs recommended reducing learning requirements to give more time for active dancing to reach training thresholds [44, 87]. Motor-skill learning demands and cognitive load will also be higher for novice dancers [28], people with poorer cognitive and physical abilities, and lower balance capabilities [28, 141–143], emphasising the potential need for more individualised programs to support both participation and health outcomes.

Variability in program success across different dance style categories and approaches suggests that program factors do contribute to disparities in health outcomes. A further meta-analysis found that dance movement therapy (DMT) produced small, consistent effects on psychological health while other dance activities were associated with larger but inconsistent effects, suggesting greater program variability [13]. DMT was also confirmed as primarily therapeutic with few motor benefits, a finding corroborated by this review for other predominately creative programs with high participant autonomy.

Although an extensive range of ‘active ingredients’ have been proposed for dance activities, only one trial compared different styles of dance programs [27], and no studies investigated the relative benefits of other program properties. Evaluations of program types and approaches, and the pathways and processes through which arts benefits health are relatively limited, but steadily gaining attention [23]. For example, a recent study examined the effects of music (vs. no music) on gait for people with Parkinson’s before and after a dance session [153], and reported that music only improved gait before a dance class, hypothesising that dancing helps participants internalise music or rhythm which supports their motor control. Experimental methods and evidence synthesis strategies may allow for greater understanding of the specific program properties that contribute most effectively to the health benefits of dance for older adults.

### Recommendations

Dance shows promise as an adaptable activity to support health and wellbeing in later life. However, health intervention studies should include sufficient details to allow reproducibility and scalability regardless of outcome measures and trial success. Inconsistent and inadequate reporting practices complicate evidence synthesis to optimise impacts and to guide future research, program design, and implementation. Additionally, study design and size did not indicate reporting quality.

#### Reporting recommendations

Golden and colleagues [32] and the NIH for music for health toolkit [119] recommended standardising reporting practices by following existing guidelines to ensure comprehensive documentation of key study aspects. Implementing frameworks such as CONSORT for RCTs, STROBE for observational studies, and TIDieR for intervention details promotes transparency, reproducibility, and research synthesis. Wholescale adoption of these guidelines by artists, practitioners, and program leaders, as well as researchers, will ensure consistent communication across disciplines and audiences.

Golden and colleagues also developed music-specific reporting recommendations based on their review and further informed by stakeholder consultation [20]. Reporting guidelines and consistent terminology for dance health [19] would be equally useful, particularly for pinpointing ‘active ingredients’. Here, we provide reporting recommendations for dance for health including key properties of programs for older people identified through this review, incorporating INNATE framework ingredients [23] and relevant advice from Golden [32], organised into TIDieR reporting categories, in S2 Table.

#### Recommendations for COS AND ‘CAIS’

We encourage the development of a core outcome set (COS) for different health domains in dance research focused on older adults, aligning with recommendations from the Golden review [32]. A COS with identified measures ensures consistency across research projects, facilitates comparisons, and improves evidence synthesis. When developed through expert consultation, including older adults with lived experience and incorporating qualitative research findings, a COS can better reflect outcome domains valued by participants [32]. The outcomes identified in our study could advance the COS development process.

Alongside a COS, we propose developing a core ‘active ingredient’ set (CAIS) to be considered and reported in each dance health study involving older people. For example, alongside COS for balance and functional mobility, a CAIS would consider movements and exercises within a dance program which challenge balance and, conceivably, classify overall program-level balance challenges as low, moderate, or high. For ingredients such as creative opportunities, levels may indicate dose. Again, a CAIS for dance health programs would be constructed through substantive stakeholder consultation, informed by the INNATE framework [23] and this review.

#### General recommendations

To enhance research translation into practice and support future studies, we offer further recommendations informed by the evidence gaps identified above, summarised in Table 9.

**Table 9:** Recommendations for future research and practice in dance for health for older adults.

Recommendations	
*For research*	*For practice*
Standardise reporting dance health trials by implementing guidelines to improve consistency and comparability.	Ensure clear and consistent documentation of participant demographics and program characteristics to facilitate cross-disciplinary communication.
Identify relevant ‘active ingredients’ and mechanisms of change to understand how dance impacts health and wellbeing, including specific pathways and processes.	Recognise how program ingredients impact participant experiences and health outcomes, and the processes (mechanisms) that drive health and behaviour change.
Conduct theory-informed research to establish clear theoretical foundations for dance program design and health and behaviour change.	Integrate theory-informed approaches to improve program delivery (e.g., motor-skill learning, locus of control, therapy frameworks, strategies to support self-efficacy and motivation).
Explore the dose-response relationship (including total dose, and dose/level of ‘active ingredients’) to determine optimal dose for different health outcomes.	Address program-related factors such as class sizes, student-teacher ratios, and volunteer or supervisor support to enhance program suitability.
Include diverse populations in research, focusing on cultural relevance and inclusivity of dance programs including equity in health and arts opportunities.	Ensure programs are culturally relevant and inclusive to facilitate participation for marginalised peoples and under-served groups.
Incorporate social determinants of health into the design, implementation, and evaluation of dance programs for older adults.	Build additional supports for physical and social activity participation for older people with restricted access to arts or community spaces.
Assess the suitability, accessibility, and inclusivity of programs for older adult participants.	Design dance programs that are flexible and adaptable to accommodate a wide range of participant needs and abilities, including those with hearing impairments, mobility limitations, and common health conditions.
	Implement remote options to improve accessibility and inclusivity (e.g. ‘Roomers’ and ‘Zoomers’ integrated dance program [140]).
Learn from older adults to understand their motivations, expectations, preferences, and ideas for dance programs and factors influencing adherence and engagement to dance, including barriers to participation and reasons for attrition.	Learn from older adults to understand their motivations, expectations, preferences, and ideas for dance programs and factors influencing adherence and engagement to dance, including barriers to participation and reasons for attrition.
Implement co-design, community consultation, and cross-disciplinary stakeholder engagement including artists and dance health practitioners to achieve a cohesive field of practice and research.	Promote and support the co-design and co-organisation of dance programs with older adults to ensure their input, experiences, and perspectives are integrated.
Investigate the differential impact of dance styles and approaches and ‘active ingredients’ on participant attendance and engagement in dance programs	Regularly assess and adapt programs based on participant feedback and engagement to improve fit and efficacy, including personal factors such as motivation, dance self-efficacy, and outcome expectations.
Evaluate the benefits of personalized and tailored dance programs compared to one-size-fits-all approaches	Where appropriate, provide individualised and progressive challenges and opportunities within dance programs to support both participation and health outcomes either by staging participants at different levels or with high teacher-student ratio to facilitate individual feedback and support
Examine the impact of facilitator expertise and training on program outcomes, and recognise, acknowledge, and incorporate practitioner-expertise	Develop training programs and resources for dance facilitators focused on skills, knowledge, and experience required to work with older adults, including guidance on adapting dance to accommodate diverse participant needs and capabilities, understanding health issues and orthopaedic limitations affecting dance capability
Investigate factors that promote participation and sustainability of dance programs for older adults in the longer term	Consider evidence-based initiatives to support program participation including social support, cognitive reframing, establishing a group identity, problem solving potential issues, and encouraging older people to prioritise activities to support health
Establish strong research to practice and practice to research pipelines, emphasising effective collaboration, co-learning and translation to support best practice in dance research and program implementation	Establish strong research to practice and practice to research pipelines, emphasising effective, collaboration, co-learning and translation to support best practice in dance research and program implementation

### Limitations

We acknowledge several limitations to our study. Data charting, collation and synthesis were compromised by poor reporting, affecting overall estimates of program types and properties. Charting and collation were iterative processes during which the extraction tool and the data categories shifted to better reflect the data. Program design and implementation descriptions were predominately text-based and scattered throughout publications. Although different research teams might have made different selections or categorisations (e.g., dance style categories, outcome domain categories), we do not expect these differences to substantively affect the results, evidence gaps or recommendations.

We intended to use categories from the INNATE framework [23], but this proved too onerous. Many INNATE ‘ingredients’ were infrequently mentioned, overlapped with other ingredients, or were difficult to place within the TIDieR framework. For the process of retrospectively identifying potential ‘active ingredients’ from research trial publications, we recommend using INNATE in the initial stages to scope potential ingredients, then to substantiate ingredients identified.

Using the percentage of positive tests in a health domain to indicate program efficacy is a somewhat blunt approach, as it does not account for effect size or negative effects (which were rare), treats all outcomes equally, and lends more weight to studies with more tests. This approach does allow comparisons across multiple domains, verified against effect sizes estimates from meta-analyses. Additionally, the tendency to run multiple tests, often without adjusting statistically, was evident in certain health categories, inflating test numbers without affecting success rates. This was particularly noticeable in trials assessing balance using 3D force platforms or brain structure and function using fMRI. For example, one study with 36 participants conducted and reported results for 60 balance tests [154], and all 131 neurological outcomes were produced across only 4 trials.

Finally, it is unknown to what degree the dance programs reviewed reflect those currently offered to older adults in the community, and the people and organisations delivering them. Some studies evaluated well-established initiatives [e.g., 42, 47, 54, 57, 60]. However, most appeared to be pilots developed for the trials, delivered by facilitators with unspecified experience working with older adults. Ongoing programs may operate differently and be more appealing as facilitators gain skills, programs are modified to suit their participants, and existing participants provide peer models, improving overall efficacy.

### Future directions

To our knowledge, this study is the first to comprehensively map and synthesise properties of arts health programs for a specific population and identify and collate potential ‘active ingredients’. Our review established that dance programs vary substantially across factors likely to differentially impact health. We used the TIDieR and INNATE frameworks and compared program success across multiple health domains. This novel approach organises and synthesises information about program factors and outcomes across many research trials to provide a unique overview of a developing knowledge area.

Combining program data and outcome data allows researchers to compare the relative success of arts health programs of different types and properties, theoretically guiding the search for ‘active ingredients’. Our review initiates this process by presenting the relative success for different styles of dance programs. Leveraging information from existing studies reduces the need for new trials, minimising cost and participant burden. Future publications will compare program success for different participant groups, program factors such as dose, and properties such as physical intensity and creative content.

We emphasise that the goal is not to redesign or reimagine dance for older people, but to incrementally shift and fine-tune program design, delivery, and evaluation to support health and social outcomes that are important and meaningful to older adults, informed by existing research and valuing and centring practitioner knowledge and expertise. For example, if physical intensity is an ‘active ingredient’ of dancing, adjusting music bpm during dance sessions may be a useful and low-cost proxy to both estimate and manipulate program intensity in the field [28]. Understanding which ingredients relate to health outcomes for specific populations will only expand and enhance the dance health practitioner’s toolbox.

### Conclusions

As a field, we have produced a substantive body of at least 115 trials of dance health programs for older adults, alongside comprehensive evidence synthesis of health outcomes. This scoping review mapped the scope, range, and effectiveness of these dance programs for older people, and identified evidence gaps and opportunities for future research and practice to support the improvement and growth of options for the use of dance as a health and social resource. To our knowledge, this is the first scoping review to comprehensively synthesise evidence of program and participant factors for any arts in health activity at this scale.

Dance programs varied substantially across factors likely to influence both participation and health outcomes, and program success varied across health domains and dance styles. The diversity of approaches and the range of health and social outcomes predicted to be impacted by dance underlines both the potential for dance to be an effective, responsive and adaptable health resource and the difficulties in comparing between studies and identifying key ‘active ingredients’ to guide future dance program development. Our results indicate that the field of dance for health—alongside arts-based approaches to health in general [32]—are not limited by a lack of research, but by issues in effectively synthesising evidence of program factors alongside health impacts.

The complexity of dance health programs necessitates comprehensive program reporting. The development of core outcomes sets for specific outcome domains matched with relevant core ‘active ingredient’ sets—COS and CAIS—would further ensure consistency across studies. Together, these approaches would improve transparency, replicability, and transferability, and support the development of theory to co-ordinate and explain the current research findings and to understand mechanisms of change and causal pathways in dance health.

Recommendations, informed by key evidence gaps and opportunities, included: 1) Establishing dance as a health and social resource for underserved groups with barriers to health, physical activity, and arts opportunities; 2) Implementation of strategies to assess program suitability and acceptability, and theory-driven approaches to support program adherence and learning; 3) Increased focus on participant and practitioner perspectives and personal factors that affect dance participation to improve dance engagement, particularly through co-design and co-production; and 4) Further mapping of ‘active ingredients’ and optimal dance dose to support health outcomes and guide program design, delivery, and evaluation. Ultimately, future research and practice must focus on understanding who our dance programs are most effective for, who they are not reaching, adequately supporting or accommodating, and how we improve accessibility, inclusivity, and tailor dance program design and implementation to optimise engagement and health impacts.

## Supporting information

S1 Table Search strategy for Medline and the PRISMA-ScR reporting checklist. (PDF)

S2 Table Reporting recommendations for dance for health (DfH) programs and research trials. (PDF)

S1 File Scoping review publications. Complete list of publications included in the scoping review (alphabetical order).
(PDF)

S2 File Basic descriptors of all trial publications included in the scoping review. (XLSX)
