# Correction: The use of dance to improve the health and wellbeing of older adults: A global scoping review of research trials

**DOI:** 10.1371/journal.pone.0322890

**Published:** 2025-04-22

**Authors:** The Staff

The hyperlink in the Data Availability statement is missing. The hyperlink is: https://research-data.westernsydney.edu.au/published/ad069cf07c7e11ef8d726d4e680f35f0/.

The publisher apologizes for the error.
