# Supplementary material for: The use of dance to improve the health and wellbeing of older adults: A global scoping review of research trials
Source: PLoS One. 2024 Oct 22;19(10):e0311889. doi: 10.1371/journal.pone.0311889 (PMC11495583; doi:10.1371/journal.pone.0311889)
Supplement: S2 Table — (PDF) [file pone.0311889.s002.pdf]

Reporting recommendations for dance for health (DfH) programs and research trials

Source materials:

- TIDieR intervention reporting guidelines and checklist (Tricco et. al, 2018)
- Reporting recommendations for music-based activities in a health context (Golden et. al, 2021; Data Sheets 3&4)
- INNATE framework for 'active ingredients' in arts in health (Warren et. al, 2022)
- Tomorrow's dance and health partnership: The need for a holistic view (Fortin, 2018)

**Categories**	**Guidelines (taken from TIDieR & Golden)**
**Golden: Standardized Reporting Guidelines**	- Use appropriate, existing reporting guidelines for study design (e.g., CONSORT for RCTs, STROBE for observational studies, CARE for case reports, etc.) See the Equator Network for details: https://www.equator-network.org/.<br>- Identify and utilise existing reporting guidelines for the conditions/populations of interest.<br>- Identify and apply the reporting guidelines for the types of data collected as part of the study (e.g., eMOOD, fMRI).
**TIDieR: Name/ Description**	Provide the name or a short phrase that describes the intervention.
**DfH prompts**	- Indicate dance style/genre or creative, mixed style, or specialist program.<br>- Include any participant characteristics specified in recruitment or for targeted groups that influence program design participation (e.g., low-mobility, frail, high fall risk, inactive, dance novice, low-income area).
**Golden: Development of Project**	- Had this intervention been previously developed or piloted? If so, by whom?<br>- Had this intervention been previously evaluated? If so, by whom? What were the results?<br>- Alternatively, was this a bespoke project/program designed specifically for a study / research endeavor? If so, who designed it? based on what research/literature?
**DfH prompts**	- Include any community, stakeholder or expert consultation undertaken.<br>- For co-designed projects, include details about how different stakeholder were involved in program conception and development.<br>- Briefly describe project management and economic resources required including any participant charges.
**TIDieR: WHY Rationale for program**	Describe any rationale, theory, or goal of the elements essential to the intervention. Include any information about program components that are theory-driven. The term "active ingredient" can be used to refer to program components specifically linked to its effect on outcomes. The known or supposed mechanism of action of the active component/s of the intervention should be described.

**DfH prompts**	- Why that specific dance program for that specific population?<br>- What needs or problems does the program meet or address (including equality, diversity and inclusion)?<br>- Describe factors determining activity selection and program/study design decisions, either previous research, theories of change, external factors (e.g. funding constraints, facilitator experience), or pre-existing dance program.<br>- Statements about program properties, active ingredients and mechanisms should be supported by prior research or verified through design or evaluation (e.g., balance challenges were provided by integrating falls prevention protocols, program assessed for enjoyment, cultural sensitivity or cultural relevance, hospitality).<br>- Describe the main program approach: Content-centred, participant-centred or mixed (Fortin, 2018), and consider further INNATE descriptions of programs as values-directed, outcomes-directed, and autonomy-directed.<br>- Key factors: Dose; style/genre; culture; program design and content; adaptations and tailoring for population; pedagogy.<br><br>Exemplar studies: Marquez 2015; Ho 2022; Ambegaonkar, 2022; Haynes, 2023; Bennett, 2018; Britten 2023; Aguinaga, 2021; Bungay 2020; Joung 2019; Rodrigues-Krause, 2018
**Golden: Population Under Study**	- Demographics: race/ethnicity; gender; age (mean (sd), min, max, categories); condition/diagnosis (if applicable); geographic region; group (if applicable; e.g., veteran, LGBTQ+, recently transitioned into age-care, low-income etc.). May also include education, socioeconomic status, language.<br>- How were participants selected for inclusion in the program or study, or how did they sign up?
**DfH prompts**	- Prior dance experience: Survey and report prior dance experience for content-centered programs where dance skill determines degree of challenge.<br>- Consider participant's impressions of dance activities before participating (e.g., safety, appropriateness).<br>- Physical activity levels: If relevant, report prior physical activity/exercise levels (using International Physical Activity Questionnaire (IPAQ) or similar).<br>- Consider social diversity of participants.
**TIDieR: WHAT MATERIALS Physical or informational materials used in intervention**	Describe any physical or informational materials used in the intervention, including those provided to participants or used in intervention delivery or in training of intervention providers and where they can be accessed (E.g. online appendix, URL). This list of materials can be regarded as comparable with the "ingredients" required for a recipe.
**DfH prompts**	- For manualised programs, provide links to manuals or exemplar sessions where possible.<br>- Other materials may include a structural plan for the program, exemplar lesson plans, materials provided for dance health practitioner and volunteer training, records of 'homework' tasks for participants, music playlists, welcome packs, support pack and instructions for participants of program delivered live, remotely.

**TIDieR: WHAT PROCEDURES Procedures, activities and processes**	Describe what processes, activities, or procedures the intervention provider/s carried out including any enabling or support activities. Continuing the recipe metaphor used above, this item refers to the "methods" section of a recipe and where intervention materials ("ingredients") are involved, describes what is to be done with them.
**DfH prompts**	WHAT PROCEDURES should describe:<br>1) Session structure and content; 2) How the content is delivered, i.e., the approach to teaching or program delivery; 3) Active ingredients.<br>- Include a plan or structure for a typical dance session in a table. Information may include class sections, section duration (mins), activities (sequences, tasks, movements), purpose of activities and/or intended outcomes..<br>- For progressive, titrated programs, consider including program overview with information on what components of the program change over time (e.g., Rodrigues-Krause, 2018), consider ongoing skill acquisition requirements.<br>- Where possible, provide links to audiovisual recordings of sessions or demonstrations of class content by facilitators or select volunteers.<br>- Describe what music is used and how it is used in the program whether live or pre-recorded, the significance to participants (e.g., from a particular era), genres/ types or variety, and pace or bpm.<br>- Describe the pedagogy and teaching approaches and how this reflects the focus of the program (see rationale).<br>ACTIVE INGREDIENTS should include:<br>1) Description of 'active ingredients'; 2) Classified as low, moderate or high challenge or dose where possible (e.g., low-creative vs. high-creative, also see TIDieR: HOW MUCH); and 3) any progression across the program<br>- Artistic and creative opportunities: Improvisation and creative tasks, focus on artistry or style (guided discovery, artistic dimensions, themes, involvement of the imagination, emotional stimuli, interpretation of music, self-expression, autonomy, choice).<br>- Neuromotor learning and co-ordination: Difficulty level of dance sequences and how is difficulty is managed (layering movement components (e.g., co-ordinate arms with legs), increasing speed or length of dance exercises, introducing more complicated movements).<br>- Cognitive challenges: Level of challenge and how challenge is managed (learning and recall (immediate or delayed), availability of movement cues, switching leader and follower roles, singing while dancing, other dual tasks).<br>- Balance challenges: Describe movements that challenge balance.<br>- Integrated and informal social exchanges: Shared focus, shared activities, social exchanges, structured social time during activity and outside activity, managing communications.<br>- Further 'active ingredients' may include: Participant choice; cultural aspects of programs; opportunities for reminiscence; formal and informal performance opportunities; hospitality (belonging) and non-hierarchical approaches to delivery and facilitation.<br><br>Exemplar studies: Hackney, 2007; Coubard 2011; Kluge, 2012; Cruz-Ferreira, 2015; Marquez, 2015; Bennett, 2017; Rodrigues-Krause, 2018; Joung, 2019; Bungay, 2020; Coelho, 2021; Kennedy, 2023; Fanning, 2023

**TIDieR: WHO PROVIDED Intervention provider's expertise, background and training given**	For each category of intervention provider, describe their expertise, background and any specific training given. Important issues to address in the description might include the number of providers involved; what pre-existing specific skills, expertise, and experience providers required; details of any additional intervention-specific training provided before and/or during the study; and assessment of competence in delivering the intervention.
**DfH prompts**	- Describe delivery model (solo facilitator, co-teaching, facilitator with trained volunteers, train the trainer) and any additional people involved.<br>- Report number of participants per class and student teacher ratios.<br>- Report facilitator experience delivering dance programs overall and for the target population
**TIDieR: HOW Modes of delivery**	Describe the modes of delivery (such as face to face or online) of the intervention.
**TIDieR: WHERE Type(s) of location(s) and necessary infrastructure**	Describe the type(s) of location(s) where the intervention occurred, including any necessary infrastructure or relevant features. Include the country, the setting, and the availability of certain facilities or equipment.
**DfH prompts**	- Location: Specify size of room required, flooring (e.g., sprung, wooden, carpeted), internal access and safety requirements.<br>- Equipment specifications (in-person): Chairs, (e.g. solid chairs with no arms and non-slip), wireless headset microphones, props, heart rate monitors, accelerometers, apps.<br>- Equipment specifications (live, remote delivery): Stable internet connection, WIFI, technical support, computers or mobile devices, safety monitoring<br>- Accessibility: Transport links, parking, local infrastructure, etc.<br>- If relevant, consider atmosphere, privacy, belonging, familiarity.
**TIDieR: WHEN AND HOW MUCH Dose, intensity, challenges**	Describe the number of times the intervention was delivered and over what period of time including the number of sessions, their schedule, and their duration, intensity or dose.
**DfH prompts**	- Dose: Session length (mins), frequency per week, duration weeks, total hrs.<br>- Intensity (level and dose): If relevant:<br>1) How is program intensity estimated/measured (Berg, heartrate monitor)? 2) How and how often is intensity monitored (Once after class, at intervals, 4 times during class once per month)? 3) Does physical intensity vary across a single session? 4) Is intensity progressive (titrated) across the program?<br>- Challenges: As intensity, if possible, classify as low, moderate or high (consider how to estimate program challenge).<br>- Artistic and creative opportunities: High-creative vs. low-creative programs, time spent on creative tasks (e.g. 5 mins vs. 60mins).
**TIDieR: TAILORING**	If the intervention was planned to be personalised, titrated or adapted, then describe what, why, when, and how. Interventions can be tailored for several reasons, such as the participant's preference, skills, or situation; or it may be

**Personalised or adapted program**	an intrinsic element of the intervention as with increasing intensity of an exercise.
**DfH prompts**	Dance programs may be fully or partially standardised or unstandardised and:<br>1) Delivered with no tailoring or adaptations; 2) Pre-existing dance programs adapted for a specific group (age, clinical condition); 3) Have separate streams delivered for different ability levels (e.g., beginners, continuers, advanced); 4) Deliver a single program stream with inbuilt difficulty levels taught concurrently (e.g., options for seated, standing with support, and standing with no support); 5) Offer adaptions to movements and sequences on a person-by-person basis; 6) Intentionally deliver ongoing adaptations and tailoring – pre-planned or in the moment – to facilitate participation and accommodate the needs and abilities of the group or in response to participants' interest and preferences.<br>NOTE: Report unintentional changes to the program under MODIFICATIONS
**TIDieR: MODIFICATIONS Program changes**	If the intervention was modified during the course of the study, describe the changes (what, why, when, and how). Unforeseen modifications to the intervention can occur during the course of the study. If this happens, explain what was modified, why and when modifications occurred, and how the modified intervention differed from the original.
**DfH prompts**	- Did program delivery follow the original plan or was any part of the delivery altered, and why (e.g., progression through the program was slower than expected so lesson plans were modified, more volunteer support required).
**TIDieR: HOW WELL (planned) Intervention adherence or fidelity**	If intervention adherence or fidelity was assessed, describe how and by whom, and if any strategies were used to maintain or improve fidelity, describe them. Fidelity refers to the degree to which an intervention happened in the way the investigators intended it to and can affect the success of an intervention. This item refers to "how well" the intervention was received or delivered.
**DfH prompts**	- Describe how program adherence (retention and attendance) will be reported (ie., who is included in the figures). Retention refers to percentage of study completers, and attendance refers to percentage of attended sessions (see Ambegaonkar, 2022). We strongly recommend reporting program attendance for all participants who attended at least 1-2 sessions including people who subsequently dropped out (ITT). Reporting of attendance for study completers only should be secondary. Also describe how dance session attendance will be monitored, and reasons for drop-outs or not attending sessions.<br>- Program adherence can also refer to the degree to which participants adhered to the program content and participated as intended, and adherence to program intensity and challenges.<br>- Acceptability can be established by program attendance and participant feedback.<br>- Program fidelity should be checked at intervals and can refer to program delivery at a single location as well as consistency of delivery across multiple locations or by multiple instructors.

	- Also describe how program safety will be established, including monitoring for adverse events.<br>- Usability should be considered for programs delivered remotely.<br><br>Exemplar studies: Ambegaonkar, 2022; Merom, 2016 (a&b); Bennett, 2017; Rodrigues-Krause, 2018; Bungay 2020, 2022; Fanning 2023
**TIDieR: HOW WELL (actual) Was intervention delivered as planned**	If intervention adherence or fidelity was assessed, describe the extent to which the intervention was delivered as planned. For various reasons, an intervention, or parts of it, might not be delivered as intended. Authors should describe the extent to which the delivered intervention varied from the intended intervention. This information can help to explain study findings, minimise errors in interpreting study outcomes, inform future modifications to the intervention, and, when fidelity is poor, can point to the need for further studies or strategies to improve fidelity or adherence. For example, there might be some aspects of the intervention that participants do not like and this could influence their adherence.
**DfH prompts**	- Reflect on how and why any changes were made to the design or delivery of the dance program, and how well the program was received by participants.<br>- Include suggestions for future program design and delivery based on participant response and feedback.<br><br>Exemplar studies: Ambegaonkar, 2022; Merom, 2016 (a&b); Bennett, 2017; Rodrigues-Krause, 2018; Bungay 2020, 2022; Fanning 2023
