# Supplementary material for: The use of dance to improve the health and wellbeing of older adults: A global scoping review of research trials
Source: PLoS One. 2024 Oct 22;19(10):e0311889. doi: 10.1371/journal.pone.0311889 (PMC11495583; doi:10.1371/journal.pone.0311889)
Supplement: S1 File — Complete list of publications included in the scoping review (alphabetical order). (PDF) [file pone.0311889.s003.pdf]

Publications

Complete list of publications included in the scoping review (alphabetical order)

Agosti, V., Coppola, S., & Vastola, R. (2021). Moving through didactic of human movement and bodily experience: A motion analysis preliminary study. *Journal of Human Sport and Exercise*, *16*, S574–S579. https://doi.org/10.14198/jhse.2021.16.Proc2.41

Aguiñaga, S., Kaushal, N., Balbim, G. M., Wilson, R. S., Wilbur, J. E., Hughes, S., Buchner, D. M., Berbaum, M., McAuley, E., Vásquez, P. M., Marques, I. G., Wang, T., & Marquez, D. X. (2022). Latin dance and working memory: The mediating effects of physical activity among middle-aged and older Latinos. *Frontiers in Aging Neuroscience*, *14*. https://www.frontiersin.org/articles/10.3389/fnagi.2022.755154

Aguiñaga, S., Marques, I. G., Kitsiou, S., Balbim, G. M., Gerber, B. S., Buchholz, S. W., Bustamante, E. E., & Marquez, D. X. (2021). BAILAMOS with mHealth technology! Improving physical activity and well-being in middle-aged and older Latinxs: A pre-post feasibility study. *Health Education & Behavior*, *48*(5), 575–583. https://doi.org/10.1177/10901981211027517

Ambegaonkar, J. P., Matto, H., Ihara, E. S., Tompkins, C., Caswell, S. V., Cortes, N., Davis, R., Coogan, S. M., Fauntroy, V. N., Glass, E., Lee, J., Baraniecki-Zwil, G., & Dhokai, N. (2022). Dance, music, and social conversation program participation positively affects physical and mental health in community-dwelling older adults: A randomized controlled trial. *Journal of Dance Medicine & Science*, *26*(4), 255–264. https://doi.org/10.12678/1089-313X.121522f

Areeudomwong, P., Saysalum, S., Phuttanurattana, N., Sripoom, P., Buttagat, V., & Keawduangdee, P. (2019). Balance and functional fitness benefits of a Thai boxing dance program among community-dwelling older adults at risk of falling: A randomized controlled study. *Archives of Gerontology and Geriatrics*, *83*, 231–238. https://doi.org/10.1016/j.archger.2019.04.010

Balazova, Z., Marecek, R., Novakova, L., Nemcova-Elfmarkova, N., Kropacova, S., Brabenec, L., Grmela, R., Vaculikova, P., Svobodova, L., & Rektorova, I. (2021). Dance intervention impact on brain plasticity: A randomized 6-month fMRI study in non-expert older adults. *Frontiers in Aging Neuroscience*, *13*(101525824), 724064. https://doi.org/10.3389/fnagi.2021.724064

Balbim, G. M., Ajilore, O. A., Erickson, K. I., Lamar, M., Aguiñaga, S., Bustamante, E. E., & Marquez, D. X. (2021). The impact of the BAILAMOS™ dance program on brain

functional connectivity and cognition in older Latino adults: A pilot study. *Journal of Cognitive Enhancement*, *5*(1), 1–14. https://doi.org/10.1007/s41465-020-00185-1

Baniqued, P. L., Gallen, C. L., Voss, M. W., Burzynska, A. Z., Wong, C. N., Cooke, G. E., Duffy, K., Fanning, J., Ehlers, D. K., Salerno, E. A., Aguiñaga, S., McAuley, E., Kramer, A. F., & D'Esposito, M. (2018). Brain network modularity predicts exercise-related executive function gains in older adults. *Frontiers in Aging Neuroscience*, *9*, 426. https://doi.org/10.3389/fnagi.2017.00426

Bennett, C. G., Angel, N., & Hackney, M. E. (2020). Mismatch between subjective and objective motor improvements with adapted tango intervention in older adults. *Physiotherapy Research International*, *25*(3), e1835. https://doi.org/10.1002/pri.1835

Bennett, C. G., & Hackney, M. E. (2018). Effects of line dancing on physical function and perceived limitation in older adults with self-reported mobility limitations. *Disability and Rehabilitation*, *40*(11), 1259–1265. https://doi.org/10.1080/09638288.2017.1294207

Borges, E. G. da S., Cader, S. A., Vale, R. G. de S., Cruz, T. H. P., Carvalho, M. C. de G. de A., Pinto, F. M., & Dantas, E. H. M. (2012). The effect of ballroom dance on balance and functional autonomy among the isolated elderly. *Archives of Gerontology and Geriatrics*, *55*(2), 492–496.

Bracco, L., Cornaro, C., Pinto-Carral, A., Koch, S. C., & Mourey, F. (2023). Tango-therapy intervention for older adults with cognitive impairment living in nursing homes: Effects on quality of life, physical abilities and gait. *International Journal of Environmental Research and Public Health*, *20*(4), Article 4. https://doi.org/10.3390/ijerph20043521

Britten, L., Addington, C., & Astill, S. (2017). Dancing in time: Feasibility and acceptability of a contemporary dance programme to modify risk factors for falling in community dwelling older adults. *BMC Geriatrics*, *17*(1), 83. https://doi.org/10.1186/s12877-017-0476-6

Britten, L., Pina, I., Nykjaer, C., & Astill, S. (2023). Dance on: A mixed-method study into the feasibility and effectiveness of a dance programme to increase physical activity levels and wellbeing in adults and older adults. *BMC Geriatrics*, *23*(1), 48. https://doi.org/10.1186/s12877-022-03646-8

Brustio, P. R., Liubicich, M. E., Chiabrero, M., & Rabaglietti, E. (2018). Dancing in the golden age: A study on physical function, quality of life, and social engagement. *Geriatric Nursing (New York, N.Y.)*, *39*(6), 635–639. https://doi.org/10.1016/j.gerinurse.2018.04.013

Bungay, H., Hughes, S., Jacobs, C., & Zhang, J. (2022). Dance for health: The impact of creative dance sessions on older people in an acute hospital setting. *Arts & Health*, *14*(1), 1–13. https://doi.org/10.1080/17533015.2020.1725072

Bungay, H., & Jacobs, C. (2020). Dance for health: The perceptions of healthcare professionals of the impact of music and movement sessions for older people in acute hospital settings. *International Journal of Older People Nursing*, *15*(4), e12342. https://doi.org/10.1111/opn.12342

Buransri, M., & Phanpheng, Y. (2021). Effects of traditional Srichiangmai dance on balance and mobility in the elderly. *Muscles, Ligaments & Tendons Journal*, *11*(2), 215–222. https://doi.org/10.32098/mltj.02.2021.02

Burzynska, A. Z., Jiao, Y., Knecht, A. M., Fanning, J., Awick, E. A., Chen, T., Gothe, N., Voss, M. W., McAuley, E., & Kramer, A. F. (2017). White matter integrity declined over 6-months, but dance intervention improved integrity of the fornix of older adults. *Frontiers in Aging Neuroscience*, *9*(101525824), 59. https://doi.org/10.3389/fnagi.2017.00059

Cepeda, C. C. P., Lodovico, A., Fowler, N., & Rodacki, A. L. F. (2015). Effect of an eight-week Ballroom dancing program on muscle architecture in older adult females. *Journal of Aging and Physical Activity*, *23*(4), 607–612. https://doi.org/10.1123/japa.2014-0101

Cheng, L., Chang, S., Gao, Z., Wang, C., Wu, C., & Yu, L. (2019). Effects of different exercise methods at the same intensity on bone mineral density of lumbar vertebrae and proximal femur in elderly women. *Medicina Dello Sport*, *72*(1), 37–46. https://doi.org/10.23736/S0025-7826.18.03432-4

Chutimakul, L., Sukonthasab, S., Kritpet, T., & Vannalee, C. (2018). Effect of modified Khon dance performance on functional fitness in older Thai persons. *Journal of Health Research*, *32*(6), 432–439. https://doi.org/10.1108/JHR-05-2018-0009

Clifford, A. M., Shanahan, J., O'Leary, H., O'Neill, D., & Ni Bhriain, O. (2019). Social dance for health and wellbeing in later life. *Complementary Therapies in Clinical Practice*, *37*, 6–10. https://doi.org/10.1016/j.ctcp.2019.07.006

Coelho, P., Marmeleira, J., Cruz-Ferreira, A., Laranjo, L., Pereira, C., & Bravo, J. (2022). Creative dance associated with traditional Portuguese singing as a strategy for active aging: A comparative cross-sectional study. *BMC Public Health*, *22*(1), 2334–2334. https://doi.org/10.1186/s12889-022-12978-4

Coogan, S. M., Dhokai, N., Baraniecki-Zwil, G., Glass, E., & Ambegaonkar, J. P. (2023). Motivation and determinants for successful engagement among community-dwelling older adults participating in a ballroom dance program. *Journal of Dance Education*, *23*(1), 57–67. https://doi.org/10.1080/15290824.2021.1896720

Coubard, O. A., Duretz, S., Lefebvre, V., Lapalus, P., & Ferrufino, L. (2011). Practice of contemporary dance improves cognitive flexibility in aging. *Frontiers in Aging Neuroscience*, *3*(101525824), 13. https://doi.org/10.3389/fnagi.2011.00013

Cruz-Ferreira, A., Marmeleira, J., Formigo, A., Gomes, D., & Fernandes, J. (2015). Creative dance improves physical fitness and life satisfaction in older women. *Research on Aging*, *37*(8), 837–855. https://doi.org/10.1177/0164027514568103

da Silva Borges, E. G., de Souza Vale, R. G., Cader, S. A., Leal, S., Miguel, F., Pernambuco, C. S., & Dantas, E. H. M. (2014). Postural balance and falls in elderly nursing home residents enrolled in a ballroom dancing program. *Archives of Gerontology and Geriatrics*, *59*(2), 312–316. https://doi.org/10.1016/j.archger.2014.03.013

Dhokai, N., Matto, H., Ihara, E. S., Tompkins, C. J., Caswell, S. V., Cortes, N., Davis, R., Coogan, S. M., Fauntroy, V. N., Glass, E., Lee, J. (Moon), Baraniecki-Zwil, G., & Ambegaonkar, J. P. (2023). Community arts engagement supports perceptions of personal growth in older adults. *Journal of Aging Studies*, *66*, 101142. https://doi.org/10.1016/j.jaging.2023.101142

Douka, S., Zilidou, V. I., Lilou, O., & Manou, V. (2019). Traditional dance improves the physical fitness and well-being of the elderly. *Frontiers in Aging Neuroscience*, *11*(101525824), 75. https://doi.org/10.3389/fnagi.2019.00075

Douka, S., Zilidou, V. I., Lilou, O., & Tsolaki, M. (2019). Greek traditional dances: A way to support intellectual, psychological, and motor functions in senior citizens at risk of neurodegeneration. *Frontiers in Aging Neuroscience*, *11*(6). https://doi.org/10.3389/fnagi.2019.00006

Ehlers, D. K., Daugherty, A. M., Burzynska, A. Z., Fanning, J., Awick, E. A., Chaddock-Heyman, L., Kramer, A. F., & McAuley, E. (2017). Regional brain volumes moderate, but do not mediate, the effects of group-based exercise training on reductions in loneliness in older adults. *Frontiers in Aging Neuroscience*, *9*(101525824), 110. https://doi.org/10.3389/fnagi.2017.00110

Ehlers, D. K., Fanning, J., Awick, E. A., Kramer, A. F., & McAuley, E. (2016). Contamination by an active control condition in a randomized exercise trial. *PLOS ONE*, *11*(10), e0164246. https://doi.org/10.1371/journal.pone.0164246

Esmail, A., Vrinceanu, T., Lussier, M., Predovan, D., Berryman, N., Houle, J., Karelis, A., Grenier, S., Minh Vu, T. T., Villalpando, J. M., & Bherer, L. (2020). Effects of dance/movement training vs. aerobic exercise training on cognition, physical fitness and quality of life in older adults: A randomized controlled trial. *Journal of Bodywork and Movement Therapies*, *24*(1), 212–220. https://doi.org/10.1016/j.jbmt.2019.05.004

Eyigor, S., Karapolat, H., Durmaz, B., Ibisoglu, U., & Cakir, S. (2009). A randomized controlled trial of Turkish folklore dance on the physical performance, balance, depression and quality of life in older women. *Archives of Gerontology and Geriatrics*, *48*(1), 84–88.

Fanning, J. T., Barnstaple, R., Babcock, P., Black, A., Collier, N., Linville, M. C., McGee, C., Morgan, A. R., Rice, P., Thomas, J. T., Thumuluri, D., Vogeley, A., Laurita-Spanglet, J., Hugenschmidt, C. E., & Soriano, C. T. (2023). Virtual delivery of improvisational movement and social engagement interventions in the IMOVE trial during the COVID-19 pandemic. *Contemporary Clinical Trials Communications*, *33*, 101102. https://doi.org/10.1016/j.conctc.2023.101102

Farmer, C., Laws, H., Eldon, S., & Maliphant, R. (2022). Relationships between dance, health and aesthetic performance in a company of mature dancers: An exploratory study. *Journal of Dance & Somatic Practices*, *14*(2), 197–216. https://doi.org/10.1386/jdsp_00084_1

Fausto, B. A., Azimipour, S., Charles, L., Yarborough, C., Grullon, K., Hokett, E., Duberstein, P. R., & Gluck, M. A. (2022). Cardio-dance exercise to improve cognition and mood in older African Americans: A propensity-matched cohort study. *Journal of Applied Gerontology*, *41*(2), 496–505. https://doi.org/10.1177/073346482110105

Federici, A., Bellagamba, S., & Rocchi, M. B. L. (2005). Does dance-based training improve balance in adult and young old subjects? A pilot randomized controlled trial. *Aging Clinical and Experimental Research*, *17*(5), 385–389.

Ferrufino, L., Bril, B., Dietrich, G., Nonaka, T., & Coubard, O. A. (2011). Practice of contemporary dance promotes stochastic postural control in aging. *Frontiers in Human Neuroscience*, *5*(101477954), 169. https://doi.org/10.3389/fnhum.2011.00169

Filar-Mierzwa, K., Dlugosz, M., Marchewka, A., Dabrowski, Z., & Poznanska, A. (2017). The effect of dance therapy on the balance of women over 60 years of age: The influence of dance therapy for the elderly. *Journal of Women & Aging*, *29*(4), 348–355. https://doi.org/10.1080/08952841.2016.1194689

Filar-Mierzwa, K., Dlugosz-Bos, M., Marchewka, A., & Aleksander-Szymanowicz, P. (2021). Effect of different forms of physical activity on balance in older women. *Journal of Women & Aging*, *33*(5), 487–502. https://doi.org/10.1080/08952841.2020.1718579

Filar-Mierzwa, K., Marchewka, A., Bac, A., Kulis, A., Dabrowski, Z., & Teleglow, A. (2017). Effects of dance therapy on the selected hematological and rheological indicators in older women. *Clinical Hemorheology and Microcirculation*, *66*(2), 157–165. https://doi.org/10.3233/CH-160241

Filar-Mierzwa, K., Marchewka, A., Dabrowski, Z., Bac, A., & Marchewka, J. (2019). Effects of dance movement therapy on the rheological properties of blood in elderly women. *Clinical Hemorheology and Microcirculation*, *72*(2), 211–219. https://doi.org/10.3233/CH-180470

Filar-Mierzwa, K., Marchewka, A., Dabrowski, Z., Wójcik, B., Superata, J., & Poznańska, A. (2014). Effects of rehabilitation in the form of dance and movement therapy on nitric

oxide levels in elderly woman. *Medicina Sportiva*, *18*(4), 130–133. https://doi.org/10.5604/17342260.1127308

Franchi, M. V., Badiali, F., Sarto, F., Muller, P., Muller, N. G., Rehfeld, K., Monti, E., Rankin, D., Longo, S., Lund, J., Hokelmann, A., & Narici, M. (2022). Neuromuscular aging: A case for the neuroprotective effects of dancing. *Gerontology*, *fpb, 7601655*, 1–9. https://doi.org/10.1159/000524843

Franco, M. R., Sherrington, C., Tiedemann, A., Pereira, L. S., Perracini, M. R., Faria, C. S. G., Negrao-Filho, R. F., Pinto, R. Z., & Pastre, C. M. (2020). Effect of senior dance (DanSE) on fall risk factors in older adults: A randomized controlled trial. *Physical Therapy*, *100*(4), 600–608. https://doi.org/10.1093/ptj/pzz187

Garcia Gouvêa, J. A., Dias Antunes, M., Bortolozzi, F., Marques, A. G., & Marques Gomes Bertolini, S. M. (2017). Impact of senior dance on emotional and motor parameters and quality of life of the elderly. *Rev Rene*, *18*(1), 51–58. https://doi.org/10.15253/2175-6783.2017000100008

Goldberg, W. G., & Fitzpatrick, J. J. (1980). Movement therapy with the aged. *Nursing Research*, *29*(6), 339–346.

Granacher, U., Muehlbauer, T., Bridenbaugh, S. A., Wolf, M., Roth, R., Gschwind, Y., Wolf, I., Mata, R., & Kressig, R. W. (2012). Effects of a salsa dance training on balance and strength performance in older adults. *Gerontology*, *58*(4), 305–312. https://doi.org/10.1159/000334814

Guzman, J., Aguinaga, S., Balbim, G. M., Lamar, M., Marques, I. G., & Marquez, D. X. (2021). The effects of the BAILAMOS dance program on hippocampal volume in older Latinos: A randomized controlled pilot study. *Translational Behavioral Medicine*, *11*(10), 1857–1862. https://doi.org/10.1093/tbm/ibab009

Hackney, M. E., Byers, C., Butler, G., Sweeney, M., Rossbach, L., & Bozzorg, A. (2015). Adapted Tango improves mobility, motor-cognitive function, and gait but not cognition in older adults in independent living. *Journal of the American Geriatrics Society*, *63*(10), 2105–2113. https://doi.org/10.1111/jgs.13650

Hackney, M. E., Kantorovich, S., & Earhart, G. M. (2007). A study on the effects of Argentine tango as a form of partnered dance for those with Parkinson Disease and the healthy elderly. *American Journal of Dance Therapy*, *29*(2), 109–127. https://doi.org/10.1007/s10465-007-9039-2

Hamacher, D., Hamacher, D., Rehfeld, K., Hokelmann, A., & Schega, L. (2015). The effect of a six-month dancing program on motor-cognitive dual-task performance in older adults. *Journal of Aging and Physical Activity*, *23*(4), 647–652. https://doi.org/10.1123/japa.2014-0067

Hamacher, D., Hamacher, D., Rehfeld, K., & Schega, L. (2016). Motor-cognitive dual-task training improves local dynamic stability of normal walking in older individuals. *Clinical Biomechanics*, *32*, 138–141. https://doi.org/10.1016/j.clinbiomech.2015.11.021

Hamburg, J., & Clair, A. A. (2003). The effects of a Laban-based movement program with music on measures of balance and gait in older adults. *Activities, Adaptation & Aging*, *28*(1), 17–33.

Hewston, P., Kennedy, C., Ioannidis, G., Merom, D., Hladysh, G., Marr, S., Lee, J., Sztramko, R., Trainor, L., Grenier, A., Woolhouse, M. H., Patterson, C., & Papaioannou, A. (2022). Development of GERAS DANcing for cognition and exercise (DANCE): A feasibility study. *Pilot and Feasibility Studies*, *8*(1), Article 1. https://doi.org/10.1186/s40814-021-00956-3

Ho, V., Li, X., & Smith, G. D. (2022). An exploratory study to assess the impact of a chair-based dance intervention among older people with depressive symptoms in residential care. *Topics in Geriatric Rehabilitation*, *38*(2), 131–139. https://doi.org/10.1097/TGR.0000000000000354

Hofgaard, J., Ermidis, G., & Mohr, M. (2019). Effects of a 6-week Faroese chain dance programme on postural balance, physical function, and health profile in elderly subjects: A pilot study. *BioMed Research International*, *2019*, 1–9. https://doi.org/10.1155/2019/5392970

Holmerova, I., Machacova, K., Vankova, H., Veleta, P., Juraskova, B., Hrnciarikova, D., Volicer, L., & Andel, R. (2010). Effect of the Exercise Dance for Seniors (EXDASE) program on lower-body functioning among institutionalized older adults. *Journal of Aging and Health*, *22*(1), 106–119. https://doi.org/10.1177/0898264309351738

Hopkins, D. R., Murrah, B., Werner, W., Hoeger, K., & Colbert Rhodes, R. (1990). Effect of low-impact aerobic dance on the functional fitness of elderly women. *The Gerontologist*, *30*(2), 189–192. https://doi.org/10.1093/geront/30.2.189

Hui, E., Chui, B. T., & Woo, J. (2009). Effects of dance on physical and psychological well-being in older persons. *Archives of Gerontology and Geriatrics*, *49*(1), e45-50. https://doi.org/10.1016/j.archger.2008.08.006

Hyodo, K., Kidokoro, T., Yamaguchi, D., Iida, M., Watanabe, Y., Ueno, A., Noda, T., Kawahara, K., Nishida, S., Kai, Y., & Arao, T. (2023). Feasibility, safety, enjoyment, and system usability of web-based aerobic dance exercise program in older adults: Single-arm pilot study. *JMIR Aging*, *6*(e39898). https://doi.org/10.2196/39898

Janyacharoen, T., Laophosri, M., Kanpittaya, J., Auvichayapat, P., & Sawanyawisuth, K. (2013). Physical performance in recently aged adults after 6 weeks traditional Thai

dance: A randomized controlled trial. *Clinical Interventions in Aging*, *8*(101273480), 855–859. https://doi.org/10.2147/CIA.S41076

Jeffrey, A., Markula, P., & Story, C. (2022). Women's articulations of aging: "Learning to be affected" through experiences in recreational ballet. *Frontiers in Sports and Active Living*, *4*(795956). https://doi.org/10.3389/fspor.2022.795956

Jong, Y.-M., Lee, M.-S., & Lee, J.-K. (2018). Effects of participation in DanceSports program on age identity, existential identity, and ego integrity of the elderly in South Korea. *Indian Journal of Public Health Research and Development*, *9*(11), 1076–1079. https://doi.org/10.5958/0976-5506.2018.01599.1

Joung, H. J., & Lee, Y. (2019). Effect of creative dance on fitness, functional balance, and mobility control in the elderly. *Gerontology*, *65*(5), 537–546. https://doi.org/10.1159/000499402

Kaewjoho, C., Mato, L., Thaweewannakij, T., Nakmareong, S., Phadungkit, S., Gaogasigam, C., & Amatachaya, S. (2020). Thai dance exercises benefited functional mobility and fall rates among community-dwelling older individuals. *Hong Kong Physiotherapy Journal*, *40*(1), 19–27. https://doi.org/10.1142/S1013702520500031

Kattenstroth, J.-C., Kalisch, T., Holt, S., Tegenthoff, M., & Dinse, H. R. (2013). Six months of dance intervention enhances postural, sensorimotor, and cognitive performance in elderly without affecting cardio-respiratory functions. *Frontiers in Aging Neuroscience*, *5*, 5. https://doi.org/10.3389/fnagi.2013.00005

Kennedy, C. C., Hewston, P., Ioannidis, G., Egbujie, B., Marr, S., Negm, A., Lee, J., Hladysh, G., Sztramko, R., Woo, T., Misiaszek, B., Patterson, C., & Papaioannou, A. (2022). Effect of the GERAS DANcing for cognition and exercise program on physical function in older adults. *Journal of Aging and Physical Activity*, *31*(2), 182–190. https://doi.org/10.1123/japa.2021-0504

Kim, S.-M., Park, H.-J., Min, B.-J., & So, W.-Y. (2018). Effects of a Korean traditional dance program on health-related fitness and blood lipid profiles in Korean elderly females. *Iranian Journal of Public Health*, *47*(1), 127–129.

Kluge, M., Tang, A., Glick, L., LeCompte, M., & Willis, B. (2012). Let's Keep Moving: A dance movement class for older women recently relocated to a continuing care retirement community (CCRC). *Arts & Health*, *4*(1), 4–15. https://doi.org/10.1080/17533015.2010.551717

Kosmat, H., & Vranic, A. (2017). The efficacy of a dance intervention as cognitive training for the old-old. *Journal of Aging and Physical Activity*, *25*(1), 32–40. https://doi.org/10.1123/japa.2015-0264

Krampe, J. (2013). Exploring the effects of dance-based therapy on balance and mobility in older adults. *Western Journal of Nursing Research*, *35*(1), 39–56. https://doi.org/10.1177/0193945911423266

Krampe, J., Miller, S. J., Echebiri, C., Rantz, M. J., & Skubic, M. (2014). Nighttime restfulness during daytime dance therapy: An exploratory study using bed sensors. *Western Journal of Nursing Research*, *36*(3), 362–373. https://doi.org/10.1177/0193945913503716

Krampe, J., Wagner, J. M., Hawthorne, K., Sanazaro, D., Wong-Anuchit, C., Budhathoki, C., Lorenz, R. A., & Raaf, S. (2014). Does dance-based therapy increase gait speed in older adults with chronic lower extremity pain: A feasibility study. *Geriatric Nursing*, *35*(5), 339–344. https://doi.org/10.1016/j.gerinurse.2014.03.008

Kropacova, S., Mitterova, K., Klobusiakova, P., Brabenec, L., Anderkova, L., Nemcova-Elfmarkova, N., Balazova, Z., Rektor, I., Grmela, R., Svobodová, L., Vaculikova, P., & Rektorova, I. (2019). Cognitive effects of dance-movement intervention in a mixed group of seniors are not dependent on hippocampal atrophy. *Journal of Neural Transmission*, *126*(11), 1455–1463. https://doi.org/10.1007/s00702-019-02068-y

Li, H., Qiu, X., Yang, Z., Zhang, Z., Wang, G., Kim, Y., & Kim, S. (2022). Effects of Cha-cha dance training on the balance ability of the healthy elderly. *International Journal of Environmental Research and Public Health*, *19*(13535). https://doi.org/10.3390/ijerph192013535

Lima, M. M. S., & Vieira, A. P. (2007). Ballroom dance as therapy for the elderly in Brazil. *American Journal of Dance Therapy*, *29*(2), 129–142. https://doi.org/10.1007/s10465-007-9040-9

Lin, L.-J., McClear, E., & Tabourne, C. E. S. (2008). The outcomes of therapeutic dance movement on physical and emotional functioning for elderly people. *American Journal of Recreation Therapy*, *7*(1), 25–34.

Lu, T., Song, Q.-H., Xu, R.-M., Guo, Y.-H., Wang, F., Hu, J.-P., Wang, Y., & Zhang, L.-Y. (2015). Dance combined with magnetic pulse stimulates the ability of walk and balance in elder people. *International Journal of Clinical and Experimental Medicine*, *8*(3), 4381–4386.

Machacova, K., Vankova, H., Volicer, L., Veleta, P., & Holmerova, I. (2017). Dance as prevention of late life functional decline among nursing home residents. *Journal of Applied Gerontology*, *36*(12), 1453–1470. https://doi.org/10.1177/0733464815602111

Marchewka, A., Filar-Mierzwa, K., Dąbrowski, Z., & Teległó, A. (2015). Effects of rhythmic exercise performed to music on the rheological properties of blood in women over 60 years of age. *Clinical Hemorheology and Microcirculation*, *60*(4), 363–373. https://doi.org/10.3233/CH-131793

Marmeleira, J. F., Pereira, C., Cruz-Ferreira, A., Fretes, V., Pisco, R., & Fernandes, O. M. (2009). Creative dance can enhance proprioception in older adults. *The Journal of Sports Medicine and Physical Fitness*, *49*(4), 480–485.

Marquez, D. X., Bustamante, E. E., Aguiñaga, S., & Hernandez, R. (2015). BAILAMOS©: Development, pilot testing, and future directions of a Latin dance program for older Latinos. *Health Education & Behavior*, *42*(5), 604–610. https://doi.org/10.1177/1090198114543006

Marquez, D. X., Wilbur, J., Hughes, S., Wilson, R., Buchner, D. M., Berbaum, M. L., McAuley, E., Aguiñaga, S., Balbim, G. M., Vásquez, P. M., Marques, I. G., Wang, T., & Kaushal, N. (2022). BAILA: A randomized controlled trial of Latin dancing to increase physical activity in Spanish-speaking older Latinos. *Annals of Behavioral Medicine*, *56*(12), 1231–1243. https://doi.org/10.1093/abm/kaac009

Marquez, D. X., Wilson, R., Aguinaga, S., Vasquez, P., Fogg, L., Yang, Z., Wilbur, J., Hughes, S., & Spanbauer, C. (2017). Regular Latin dancing and health education may improve cognition of late middle-aged and older Latinos. *Journal of Aging and Physical Activity*, *25*(3), 482–489. https://doi.org/10.1123/japa.2016-0049

Martin-Wylie, E., Urmston, E., & Redding, E. (2022). Impact of creative dance on subjective well-being amongst older adults: An arts-informed photo-elicitation study. *Arts & Health*, *16*(1), 1–17. https://doi.org/10.1080/17533015.2022.2156562

Matto, H., Ihara, E., Tompkins, C., McNeil, K., Lopez-Piper, A., Eber, M., Dhokai, N., Davis, R., Cortes, N., Caswell, S., Coogan, S., Fauntroy, V., Glass, E., Baraniecki-Zwil, G., & Ambegaonkar, J. (2021). A novel participant-empowered pedagogical approach to engage and retain control group participants in arts-based randomized controlled trials. *Families in Society: The Journal of Contemporary Social Services*, *102*(4), 529–537. https://doi.org/10.1177/1044389421997356

Mavrovouniotis, F. H., Argiriadou, E. A., & Papaioannou, C. S. (2010). Greek traditional dances and quality of old people's life. *Journal of Bodywork & Movement Therapies*, *14*(3), 209–218. https://doi.org/10.1016/j.jbmt.2008.11.005

McKinley, P., Jacobson, A., Leroux, A., Bednarczyk, V., Rossignol, M., & Fung, J. (2008). Effect of a community-based Argentine tango dance program on functional balance and confidence in older adults. *Journal of Aging and Physical Activity*, *16*(4), 435–453.

Mendez Colmenares, A., Voss, M. W., Fanning, J., Salerno, E. A., Gothe, N. P., Thomas, M. L., McAuley, E., Kramer, A. F., & Burzynska, A. Z. (2021). White matter plasticity in healthy older adults: The effects of aerobic exercise. *NeuroImage*, *239*(cpp, 9215515), 118305. https://doi.org/10.1016/j.neuroimage.2021.118305

Meng, X., Li, G., Zhang, G., Yin, H., Jia, Y., Wang, S., Shang, B., Wang, C., & Chen, L. (2020). Effects of dance intervention on frailty among older adults. *Archives of Gerontology and Geriatrics*, *88*(8214379, 7ax), 104001. https://doi.org/10.1016/j.archger.2019.104001

Merom, D., Grunseit, A., Eramudugolla, R., Jefferis, B., Mcneill, J., & Anstey, K. J. (2016). Cognitive benefits of social dancing and walking in old age: The Dancing Mind randomized controlled trial. *Frontiers in Aging Neuroscience*, *8*, 26. https://doi.org/10.3389/fnagi.2016.00026

Merom, D., Mathieu, E., Cerin, E., Morton, R. L., Simpson, J. M., Rissel, C., Anstey, K. J., Sherrington, C., Lord, S. R., & Cumming, R. G. (2016). Social dancing and incidence of falls in older adults: A cluster randomised controlled trial. *PLOS MEDICINE*, *13*(8), e1002112. https://doi.org/10.1371/journal.pmed.1002112

Minsterova, A. S., Klobusiakova, P., Kropacova, S., Novakova, L., Brabenec, L., Balazova, Z., Grmela, R., Skotakova, A., Svobodova, L., & Rektorova, I. (2020). Multishell diffusion MRI reflects improved physical fitness induced by dance intervention. *Neural Plasticity*, *2020*, 1–9. https://doi.org/10.1155/2020/8836925

Mitterova, K., Klobusiakova, P., Sejnoha Minsterova, A., Kropacova, S., Balazova, Z., Tocik, J., Vaculikova, P., Skotakova, A., Grmela, R., & Rektorova, I. (2021). Impact of cognitive reserve on dance intervention-induced changes in brain plasticity. *Scientific Reports*, *11*(1), 18527. https://doi.org/10.1038/s41598-021-97323-2

Muller, P., Rehfeld, K., Schmicker, M., Hokelmann, A., Dordevic, M., Lessmann, V., Brigadski, T., Kaufmann, J., & Muller, N. G. (2017). Evolution of neuroplasticity in response to physical activity in old age: The case for dancing. *Frontiers in Aging Neuroscience*, *9*(101525824), 56. https://doi.org/10.3389/fnagi.2017.00056

Noopud, P., Suputtitada, A., Khongprasert, S., & Kanungsukkasem, V. (2019). Effects of Thai traditional dance on balance performance in daily life among older women. *Aging Clinical and Experimental Research*, *31*(7), 961–967. https://doi.org/10.1007/s40520-018-1040-8

Nur, K. R. M., Susanto, T., Yunanto, R. A., Susumaningrum, L. A., & Rasni, H. (2022). Traditional dance “Molong Kopi” for maintaining of health status among older adults in long-term care of Indonesia. *Working with Older People*, *26*(3), 238–245. https://doi.org/10.1108/WWOP-04-2021-0017

Oliveira, B. R. R., Matos, I. C., Maranhão Neto, G. A., Rodrigues, F., Monteiro, D., Lattari, E., & Machado, S. (2021). A 16-week intervention on mood and life quality in elderly: Testing two exercise programs. *Cuadernos de Psicología Del Deporte*, *21*(2), 24–31.

Osgood, N. J., Meyers, B. S., & Orchowsky, S. (1990). The impact of creative dance and movement training on the life satisfaction of older adults: An exploratory study.

Journal of Applied Gerontology, *9*(3), 255–265. https://doi.org/10.1177/073346489000900302

Overdorf, V., Kollia, B., Makarec, K., & Alleva Szeles, C. (2016). The relationship between physical activity and depressive symptoms in healthy older women. *Gerontology & Geriatric Medicine*, *2*(101662571), 2333721415626859. https://doi.org/10.1177/2333721415626859

Pacheco, E., Hoyos, D. P., Watt, W. J., Lema, L., & Arango, C. M. (2016). Feasibility study: Colombian Caribbean folk dances to increase physical fitness and health-related quality of life in older women. *Journal of Aging and Physical Activity*, *24*(2), 284–289. https://doi.org/10.1123/japa.2015-0012

Paglione, V., Kenny, S. J., McDonough, M. H., Din, C., & White, K. (2023). Movement, music, and connection: Older adults' experiences of community dance. *Activities, Adaptation & Aging*, 1–23. https://doi.org/10.1080/01924788.2023.2191097

Paglione, V., Magrath, J., McDonough, M. H., Din, C., & Kenny, S. J. (2023). 'Promoting wellness, having fun, and creating community': A dance instructor's pedagogical practices and perspectives on the influence of community dance classes for older adults. *Research in Dance Education*, 1–18. https://doi.org/10.1080/14647893.2022.2159359

Pilch, W. B., Mucha, D. M., Pałka, T. A., Suder, A. E., Piotrowska, A. M., Tyka, A. K., Tota, Ł. M., & Ambrozy, T. (2015). The influence of a 12-week program of physical activity on changes in body composition and lipid and carbohydrate status in postmenopausal women. *Menopause Review (Przeglad Menopauzalny)*, *14*(4), 231–237. https://doi.org/10.5114/pm.2015.56311

Pitluk Barash, M., Shuper Engelhard, E., & Elboim-Gabyzon, M. (2023). Feasibility and effectiveness of a novel intervention integrating physical therapy exercise and Dance Movement Therapy on fall risk in community-dwelling older women: A randomized pilot study. *Healthcare*, *11*(8), 1104. https://doi.org/10.3390/healthcare11081104

Pope, J., Helwig, K., Morrison, S., Estep, A., Caswell, S., Ambegaonkar, J., & Cortes, N. (2019). Multifactorial exercise and dance-based interventions are effective in reducing falls risk in community-dwelling older adults: A comparison study. *Gait & Posture*, *70*(9416830, dcm), 370–375. https://doi.org/10.1016/j.gaitpost.2019.03.030

Purkart, B., Bertoncelj, B., Podlogar, A., & Pavletic, M. S. (2023). Improving postural stability in active older adults: Argentine Tango dance as an alternative fall-prevention strategy. *Alternative Therapies in Health & Medicine*, *29*(5), 201–209.

Rehfeld, K., Luders, A., Hokelmann, A., Lessmann, V., Kaufmann, J., Brigadski, T., Muller, P., & Muller, N. G. (2018). Dance training is superior to repetitive physical exercise in

inducing brain plasticity in the elderly. *PLOS ONE*, *13*(7), e0196636. https://doi.org/10.1371/journal.pone.0196636

Rehfeld, K., Muller, P., Aye, N., Schmicker, M., Dordevic, M., Kaufmann, J., Hokelmann, A., & Muller, N. G. (2017). Dancing or fitness sport? The effects of two training programs on hippocampal plasticity and balance abilities in healthy seniors. *Frontiers in Human Neuroscience*, *11*(101477954), 305. https://doi.org/10.3389/fnhum.2017.00305

Rektorova, I., Klobusiakova, P., Balazova, Z., Kropacova, S., Sejnoha Minsterova, A., Grmela, R., Skotakova, A., & Rektor, I. (2020). Brain structure changes in nondemented seniors after six-month dance-exercise intervention. *Acta Neurologica Scandinavica*, *141*(1), 90–97. https://doi.org/10.1111/ane.13181

Rodacki, A. L. F., Cepeda, C. P. C., Lodovico, A., & Ugrinowitsch, C. (2017). The effects of a dance-based program on the postural control in older women. *Topics in Geriatric Rehabilitation*, *33*(4), 244–249. https://doi.org/10.1097/TGR.0000000000000166

Rodrigues-Krause, J., Farinha, J. B., Ramis, T. R., Macedo, R. C. O., Boeno, F. P., Dos Santos, G. C., Vargas, J. J., Lopez, P., Grazioli, R., Costa, R. R., Pinto, R. S., Krause, M., & Reischak-Oliveira, A. (2018). Effects of dancing compared to walking on cardiovascular risk and functional capacity of older women: A randomized controlled trial. *Experimental Gerontology*, *114*, 67–77. https://doi.org/10.1016/j.exger.2018.10.015

Rodziewicz-Flis, E. A., Kawa, M., Kaczor, J. J., Szaro-Truchan, M., Flis, D. J., Lombardi, G., & Ziemann, E. (2023). Changes in selected exerkines concentration post folk-dance training are accompanied by glucose homeostasis and physical performance improvement in older adults. *Scientific Reports*, *13*(1), 8596. https://doi.org/10.1038/s41598-023-35583-w

Rodziewicz-Flis, E. A., Kawa, M., Skrobot, W. R., Flis, D. J., Wilczynska, D., Szaro-Truchan, M., Bolek-Adamek, J., & Kaczor, J. J. (2022). The positive impact of 12 weeks of dance and balance training on the circulating amyloid precursor protein and serotonin concentration as well as physical and cognitive abilities in elderly women. *Experimental Gerontology*, *162*, 111746. https://doi.org/10.1016/j.exger.2022.111746

Sampoon, K., Posri, N., & Kittichotpanich, B. (2019). Application of social dance exercise and social support program to improve quality of life for Thai older adults. *Journal of Health Research*, *33*(3), 260–266. https://doi.org/10.1108/JHR-08-2018-0071

Sawami, K., Kimura, M., Kitamura, T., Kawaguchi, M., Furusumi, M., Suishu, C., Morisaki, N., & Hattori, S. (2020). The effect of cognitive dance therapy as dementia prevention. *International Medicine*, *1*(3), 140–146. https://doi.org/10.5455/im.302644235

Serra, M. M., Alonso, A. C., Peterson, M., Mochizuki, L., Greve, J. M. D., & Garcez-Leme, L. E. (2016). Balance and muscle strength in elderly women who dance Samba. *PLOS ONE*, *11*(12), e0166105. https://doi.org/10.1371/journal.pone.0166105

Serrano-Guzman, M., Aguilar-Ferrandiz, M. E., Valenza, C. M., Ocana-Peinado, F. M., Valenza-Demet, G., & Villaverde-Gutierrez, C. (2016). Effectiveness of a flamenco and sevillanas program to enhance mobility, balance, physical activity, blood pressure, body mass, and quality of life in postmenopausal women living in the community in Spain: A randomized clinical trial. *Menopause*, *23*(9), 965–973. https://doi.org/10.1097/GME.0000000000000652

Shigematsu, R., Chang, M., Yabushita, N., Sakai, T., Nakagaichi, M., Nho, H., & Tanaka, K. (2002). Dance-based aerobic exercise may improve indices of falling risk in older women. *Age and Ageing*, *31*(4), 261–266.

Silva, K. M. D., Nitschke, R. G., Durand, M. K., Heidemann, I. T. S. B., Tholl, A. D., Rumor, P. C. F., & Moncada, M. J. A. (2022). Circle dance: Integrative and complementary practice in the daily health promotion for older adults. *Revista Brasileira de Enfermagem*, *75 (suppl 4)*(e20210003), 1–8. https://doi.org/10.1590/0034-7167-2021-0003

Sofianidis, G., Dimitriou, A.-M., & Hatzitaki, V. (2017). A comparative study of the effects of Pilates and Latin dance on static and dynamic balance in older adults. *Journal of Aging and Physical Activity*, *25*(3), 412–419. https://doi.org/10.1123/japa.2016-0164

Sofianidis, G., Hatzitaki, V., Douka, S., & Grouios, G. (2009). Effect of a 10-week traditional dance program on static and dynamic balance control in elderly adults. *Journal of Aging and Physical Activity*, *17*(2), 167–180.

Sohn, J., Park, S.-H., & Kim, S. (2018). Effects of DanceSport on walking balance and standing balance among the elderly. *Technology and Health Care*, *26*(S1), S481–S490. https://doi.org/10.3233/THC-174760

Stonnington, C. M., Krell-Roesch, J., Locke, D. E. C., Hentz, J. G., Dueck, A. C., Geda, Y. E., Tariot, P. N., & Caselli, R. J. (2019). Impact of Zumba on cognition and quality of life is independent of APOE4 carrier status in cognitively unimpaired older women: A 6-month randomized controlled pilot study. *American Journal of Alzheimer's Disease and Other Dementias*, *35*, 1–10.

Tantiwiboonchai, N., & Kritpet, T. (2017). Effects of Muay Thai aerobic dance on biochemical bone markers and physical fitness in elderly women. *Journal of Exercise Physiology Online*, *20*(1), 188–199.

Thornberg, K., Lindquist, I., & Josephsson, S. (2012). Experiences of healthy elderly participating in a creative dance workshop. *Advances in Physiotherapy*, *14*(2), 71–77. https://doi.org/10.3109/14038196.2012.662696

Vaccaro, M. G., Izzo, G., Ilacqua, A., Migliaccio, S., Baldari, C., Guidetti, L., Lenzi, A., Quattrone, A., Aversa, A., & Emerenziani, G. P. (2019). Characterization of the effects of a six-month dancing as approach for successful aging. *International Journal of Endocrinology*, *2019*(101516376), 2048391. https://doi.org/10.1155/2019/2048391

Valentine-Garzon, M. A., Maynard, M., & Selznick, S. Z. (1993). ROM dance program effects on frail elderly women in an adult day-care center. *Physical & Occupational Therapy in Geriatrics*, *11*(1), 63–83.

Vankova, H., Holmerova, I., Machacova, K., Volicer, L., Veleta, P., & Celko, A. M. (2014). The effect of dance on depressive symptoms in nursing home residents. *JAMDA*, *15*(8), 1–6. https://doi.org/10.1016/j.jamda.2014.04.013

Voss, M. W., Sutterer, M., Weng, T. B., Burzynska, A. Z., Fanning, J., Salerno, E., Gothe, N. P., Ehlers, D. K., McAuley, E., & Kramer, A. F. (2019). Nutritional supplementation boosts aerobic exercise effects on functional brain systems. *Journal of Applied Physiology*, *126*(1), 77–87. https://doi.org/10.1152/japplphysiol.00917.2017

Vrinceanu, T., Esmail, A., Berryman, N., Predovan, D., Vu, T. T. M., Villalpando, J. M., Pruessner, J. C., & Bherer, L. (2019). Dance your stress away: Comparing the effect of dance/movement training to aerobic exercise training on the cortisol awakening response in healthy older adults. *Stress*, *22*(6), 687–695. https://doi.org/10.1080/10253890.2019.1617690

Wang, L., Guo, F., Zhao, C., Zhao, M., Zhao, C., Guo, J., Zhang, L., Zhang, L., Li, Z., & Zhu, W. (2023). The effect of aerobic dancing on physical fitness and cognitive function in older adults during the COVID-19 pandemic—A natural experiment. *Sports Medicine and Health Science*, *5*(3), 196–204. https://doi.org/10.1016/j.smhs.2023.07.005

Wang, Q., & Zhao, Y. (2021). Effects of a modified Tap dance program on ankle function and postural control in older adults: A randomized controlled trial. *International Journal of Environmental Research and Public Health*, *18*(12). https://doi.org/10.3390/ijerph18126379

Waugh, M., Grunseit, A. C., Mathieu, E., & Merom, D. (2023). The psychometric properties of a novel task-based dance self-efficacy measure for older adult dance program participants. *Arts & Health*, 1–20. https://doi.org/10.1080/17533015.2021.1968446

Weighart, H., & Dipasquale, S. (2020). Insights on ten weeks of classical Ballet training and postural stability in older adults. *International Journal of Exercise Science*, *13*(1), 101–112.

Woloszyn, N., Wisniowska-Szurlej, A., Grzegorczyk, J., & Kwolek, A. (2021). The impact of physical exercises with elements of dance movement therapy on the upper limb grip strength and functional performance of elderly wheelchair users living in nursing

homes—A randomized control trial. *BMC Geriatrics*, *21*(1), 423. https://doi.org/10.1186/s12877-021-02368-7

Wu, H. Y., Tu, J. H., Hsu, C. H., & Tsao, T. H. (2016). Effects of low-impact dance on blood biochemistry, bone mineral density, the joint range of motion of lower extremities, knee extension torque, and fall in females. *Journal of Aging and Physical Activity*, *24*(1), 1–7. https://doi.org/10.1123/japa.2014-0088

Wu, V. X., Yap, X. Y., Tam, W. S. W., Goh, J., Mok, W. Y. J., & Ramazanu, S. (2023). Qualitative inquiry of a community dance program for older adults in Singapore. *Nursing & Health Sciences*, *25*(3), 341–353. https://doi.org/10.1111/nhs.13032

Wu, W.-L., Wei, T.-S., Chen, S.-K., Chang, J.-J., Guo, L.-Y., & Lin, H.-T. (2010). The effect of Chinese Yuanji-dance on dynamic balance and the associated attentional demands in elderly adults. *Journal of Sports Science and Medicine*, *9*(1), 119–126.

Zhang, X., Ni, X., & Chen, P. (2014). Study about the effects of different fitness sports on cognitive function and emotion of the aged. *Cell Biochemistry and Biophysics*, *70*(3), 1591–1596. https://doi.org/10.1007/s12013-014-0100-8

Zhang, X., Van Der Schans, C. P., Liu, Y., Krijnen, W. P., & Hobbelen, J. S. M. (2023). Efficacy of dance intervention for improving frailty among Chinese older adults living in the community: A randomized controlled trial. *Journal of Aging and Physical Activity*, *31*(5), 806–814. https://doi.org/10.1123/japa.2021-0500

Zhao, X., Qi, N., Long, H., & Yang, S. (2022). The impact of national music activities on improving long-term care for happiness of elderly people. *Frontiers in Psychology*, *13*(1009811). https://doi.org/10.3389/fpsyg.2022.1009811

Zilidou, V. I., Frantzidis, C. A., Romanopoulou, E. D., Paraskevopoulos, E., Douka, S., & Bamidis, P. D. (2018). Functional re-organization of cortical networks of senior citizens after a 24-week traditional dance program. *Frontiers in Aging Neuroscience*, *10*(101525824), 422. https://doi.org/10.3389/fnagi.2018.00422

Zygmont, A., Doliński, W., Zawadzka, D., & Pezdek, K. (2023). Uplifted by dancing community: From physical activity to well-being. *International Journal of Environmental Research and Public Health*, *20*(4), 3535. https://doi.org/10.3390/ijerph20043535
